# Distinct Cytokine Responses in Central and Systemic Compartments after Subarachnoid Haemorrhage

**DOI:** 10.1007/s12975-025-01348-y

**Published:** 2025-03-25

**Authors:** Soham Bandyopadhyay, Ben Gaastra, Ardalan Zolnourian, Patrick Garland, Chieh-Hsi Wu, Ian Galea, Diederik Bulters

**Affiliations:** 1 Clinical Neurosciences, Clinical & Experimental Sciences, Faculty of Medicine, University of Southampton, Southampton, Hampshire UK; 2 Wessex Neurological Centre, University Hospital Southampton NHS Foundation Trust, Southampton, UK; 3 School of Mathematical Sciences, University of Southampton, Southampton, UK; 4 Institute for Life Sciences, University of Southampton, Southampton, UK

**Keywords:** Subarachnoid Haemorrhage, Cytokines, Inflammation, Cerebrospinal Fluid, Blood Brain Interface, Vasospasm

## Abstract

Introduction: Neuroinflammation may contribute to outcomes following subarachnoid haemorrhage (SAH). Human cerebrospinal fluid (CSF) cytokine data is limited and its relationship with systemic inflammation is unknown. This study compares the inflammatory responses in CSF and plasma compartments, and their associations with outcome. Methods: Ten cytokines were measured in CSF and plasma from 98 SAH patients and 18 control patients. Outcome was assessed with the modified Rankin scale (mRS) and Subarachnoid Haemorrhage Outcome Tool (SAHOT) at days 7, 28, 90 and 180. Regression analyses and principal component analysis (PCA) were performed. Results: Median levels of all CSF cytokines and plasma IL-6 were higher in SAH patients than controls (p < 0.001). Plasma IL-6 peaked earlier (3 days after SAH) than CSF cytokines (7–9 days after SAH). On day 7, CSF levels were greater than plasma levels for all cytokines (p < 0.001). There was no correlation between individual cytokines in the plasma and CSF. Only plasma IL-6 levels correlated with long-term outcome (mRS (p = 0.009) and SAHOT (p = 0.007) at day 180), accounting for WFNS and blood volume. Seven principal components of cytokines had an eigenvalue greater than 1. Only the first plasma principal component (dominated by IL-6, IL-8, IL-12, IL-13, and TNF-α) was associated with outcomes (p < 0.05). Mediation analysis suggested the effects of WFNS and blood volume on outcome were not mediated by IL-6 or this principal component. Conclusion: SAH provokes an inflammatory response in CSF and plasma. The response pattern is different and distinct in each compartment. Each compartment’s relationship with outcomes differ, suggesting separate roles in SAH pathophysiology. Plasma IL-6 is independently associated with outcomes.

**Supplementary Information:**

The online version contains supplementary material available at 10.1007/s12975-025-01348-y.

## Introduction

Spontaneous subarachnoid haemorrhage (SAH) is a life-threatening and debilitating type of stroke, predominantly caused by the rupture of an intracranial aneurysm [1]. Approximately one-third of individuals who develop SAH die [2–4], and survivors often face significant long-term physical, cognitive, and functional deficits [5–11].

Neuroinflammation may be a potential mechanism leading to unfavourable outcomes post-SAH [12]. Elevated levels of cytokines in the blood and cerebrospinal fluid (CSF) have been reported in SAH patients [13–16]. Several publications highlight a link between increased cytokine levels and short-term adverse outcomes, such as delayed cerebral ischaemia (DCI) [15, 17, 18]. The most compelling evidence relates to interleukin-6 (IL-6), which has been found to rise post-SAH; elevated IL-6 levels have also been associated with DCI [15, 19, 20]. Similarly, increased levels of tumour necrosis factor-alpha (TNF-α) and IL-1 receptor antagonist (IL-1RA) have been detected in the CSF of SAH patients, and a positive correlation between these cytokines and poor outcome has been noted [18, 21, 22].

However, most of these studies were small, based on highly selected populations (poor grade SAH patients with ventriculostomy from single centres), examined single cytokines or limited panels, and were relatively selective in results reported (often without correcting for multiple comparisons). Therefore, a complete picture of the inflammatory response after SAH is still lacking. In many cases where limited data is available plasma has been used as a surrogate for CSF, an untested assumption in the absence of paired comparisons of the plasma and CSF inflammatory response and their relationship with outcome.

We, therefore, set out to 1) measure the CSF and plasma cytokine responses in a population of patients with both good and poor grade spontaneous SAH (both aneurysmal and non-aneurysmal), 2) compare the relative level and time course of cytokines in these two compartments, 3) examine their influence on each other, 4) assess their relative contribution to outcome, and 5) to what degree their relationship with outcome is through mediating the effects of blood volume and WFNS.

## Methods

### Study Design and Participants

**Fig. 1:**
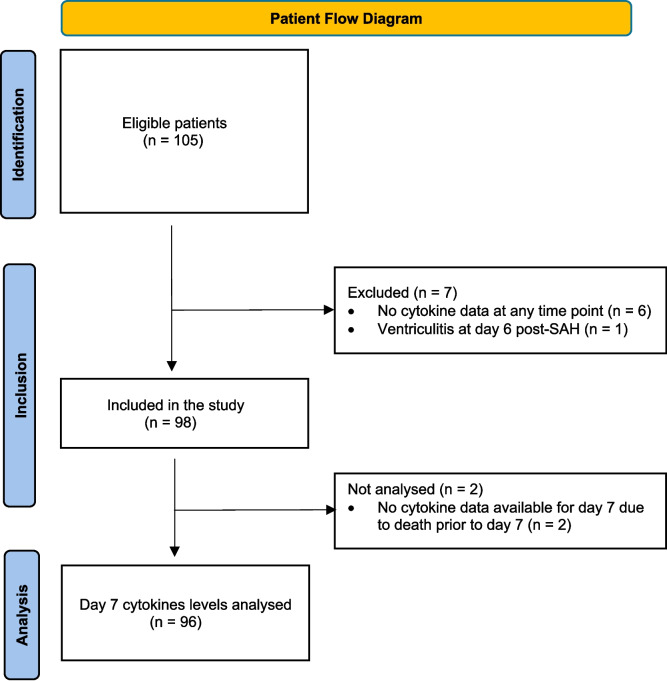
Patient flow diagram of participant inclusion and exclusion

The samples and data used in this study were obtained from a prospective, multicentre, randomised, double-blind, placebo-controlled study designed to investigate the safety and efficacy of SFX-01 (a synthetic sulforaphane stabilized within an α-cyclodextrin complex) in patients with SAH (NCT02614742). The SFX-01 after SAH (SAS) trial [23] was approved by the National Research Ethics Service South Central Hampshire A committee (ref no 16/SC/0019; ERGO 91491), and all patients or their legal representative gave consent to storage and use of their samples and data for research. The present work investigates the cytokine response after SAH using samples and data from this trial. Clinical presentation and outcome data was present for 105 patients, who were originally recruited for the trial within 48 h of sustaining Fisher grade 3 or 4 SAH. Of these 105 patients, 98 patients had data available for cytokine analysis (Fig. 1). Further CSF and plasma samples were obtained from 18 control patients without SAH participating in observational studies (National Health Service regional ethics committee approvals 11/SC/0204 and 10/H0502/53). These individuals were selected as controls as they had CSF with normal constituents obtained from either 1) a clinically indicated lumbar puncture (LP) and were subsequently found not to have an underlying inflammatory, neurodegenerative, or vascular neurological condition, or 2) a spinal anaesthetic during elective orthopaedic joint surgery.

### CSF and Plasma Sampling

Of the 98 SAH patients included in this study, 83 had CSF samples and 94 had plasma samples collected at day 7 (± 1 day) post-SAH, with 81 patients having paired day 7 CSF and plasma samples. Of the 83 patients with CSF samples, 32 had CSF samples obtained via an external ventricular drain (EVD) sited for clinical hydrocephalus. The first 3 ml of CSF (representing dead space) was discarded to ensure fresh CSF was obtained. The other 51 patients (without an EVD) had CSF available for cytokine analysis from a LP at day 7 (± 1 day). Patients with EVDs had additional alternate day CSF and plasma sampling, resulting in an additional 112 paired CSF and plasma samples, bringing the total to 193 paired CSF and plasma samples. Control patients only had one pair of spinal CSF and plasma available for cytokine analysis.

### CSF and Plasma Cytokine Analysis

Cytokines – IL-1beta (IL-1β), IL-2, IL-4, IL-6, IL-8, IL-10, IL-12, IL-13, interferon-gamma (IFN-γ), and TNF-α – were quantified using the Meso Scale Discovery (MSD) Multi-Spot Assay System (V-PLEX Proinflammatory Panel 1 Human Kit, catalogue number K15049D, Meso Scale Diagnostics headquarters is located at, Rockville, Maryland US) [24]. These cytokines were part of a commercially available panel, which provided the best balance of pro- and anti-inflammatory cytokines, while including IL-1, IL-6, IL-8 and TNF-α which have previously been investigated in various neurological disorders due to their significant roles in mediating inflammatory responses [25–27]. For instance, IL-6 levels have been found to rise over time following SAH, and higher IL-6 levels have been associated with DCI [15, 19, 20]. Similarly, increased TNF-α levels and IL-1Ra have been detected in the CSF of SAH patients, and research indicates a positive correlation between these cytokine levels and poor outcomes [18, 21, 22]. Although IL-8 levels are elevated in SAH, the association between this cytokine and unfavourable outcomes remains unclear [20, 28]. All reagents were equilibrated to room temperature prior to the assay. Calibration solutions were prepared through a series of four-fold dilution steps, and a zero calibrator was also established. CSF and plasma samples (diluted in two by an equal volume of deionised water) and calibration solutions were pipetted onto MSD plates, which were subsequently incubated at room temperature with shaking for two hours. Following this initial incubation, plates were washed, and a detection antibody solution was added. Plates were then incubated again at room temperature with shaking for another two hours. After the second incubation, plates were washed and read on the MSD instrument. Concentrations were determined based on calibration curves generated from the known calibrators.

### CSF/serum Albumin Quotient

Albumin in matched CSF and serum samples was measured on a Beckman Coulter clinical chemistry analyser using a timed endpoint method assay which relies on the reaction of albumin with Bromocresol Purple to form a coloured complex; absorbance was measured at 600 nm. The CSF/serum albumin quotient (qAlb) was calculated as a proxy measure for CSF albumin provenance, whether across the blood–brain interface (BBI) or directly as a result of the bleed [29, 30].

### CT blood volume quantification

Blood clot volume on the admission computed tomography (CT) was quantified using MIPAV (Medical Image Processing, Imaging and Visualization) v7.2 [31]. The CT image signal intensity threshold was set between 50 and 80 Hounsfield units and converted to a binary mask. Regions of interest representing blood clot were drawn manually on each slice and summed into single 3-dimensional volumes.

### Clinical Data and Outcome Measures

The following baseline variables were prospectively collected and available for analysis for all SAH participants: age, sex, race, premorbid history of hypertension, location of aneurysm (if present), method of securing aneurysm, and WFNS grade. In addition, occurrence and timing of any infection (including ventriculitis) were recorded.

Whilst inpatients, patients underwent transcranial doppler ultrasound recordings (TCDs) on alternate days and were monitored clinically for delayed cerebral ischemia (DCI). Vasospasm was defined as a mean flow velocity (MFV) of 120 cm per second (cm/s) or greater on TCDs. DCI was defined as the occurrence of focal neurological impairment (such as hemiparesis, aphasia, apraxia, hemianopia, or neglect), or a decrease of at least 2 points on the Glasgow Coma Scale (either on the total score or on one of its individual components [eye, motor on either side, verbal]) that lasted for at least 1 h, is not apparent immediately after aneurysm occlusion, and cannot be attributed to other causes by means of clinical assessment, CT or MRI scanning of the brain, and appropriate laboratory studies [32]. Functional outcomes were assessed at days 28, 90, and 180 with the modified Rankin Scale (mRS) [33] and SAH Outcome Tool (SAHOT) [34]. The mRS was also assessed on day 7. SAHOT raw scores were transformed to ordinal categories using the nomogram from the previously published Rasch analysis [34].

### Statistical Methods

All primary analyses were performed on day 7 samples. Data from other timepoints are otherwise only presented graphically. Descriptive statistics were used to summarise demographic and clinical characteristics and outcomes. Variables were tested for normality with the Shapiro-Wilks test. Means and confidence intervals are reported for parametric data. Medians and interquartile ranges (IQR) are reported for non-parametric data. The Wilcoxon-Signed Ranked test was used to compare medians between data that came from the same population. The Kruskal–Wallis test and Dunn’s post hoc test were used to compare medians between independent groups. The Bonferroni method was used to adjust for multiple comparisons when analysing associations with multiple cytokines. Any significant comparisons which did not survive correction for multiple comparisons are presented in brackets after the main result. Comparison of proportions between groups was made with Fisher’s exact test. Pearson correlation coefficients were calculated to determine correlation between different variables on a population level. A within-subjects analysis of variance followed by Tukey’s pairwise comparisons was utilised to examine differences in cytokines levels over time: day 0 (represents the onset of subarachnoid haemorrhage), day 1, day 3, day 5, day 7, day 9, day 11, and day 13.

Linear regressions were used to determine the relationship between continuous variables. Univariable logistic regression was used to examine the association between death/DCI and patient characteristics or cytokines. The mRS and SAHOT were analysed using ordinal logistic regression. Multivariable regression was used to examine the association between each outcome and each cytokine after adjusting for either 1) other cytokines, 2) blood volume, 3) WFNS grade, or 4) other cytokines, blood volume, and WFNS grade. The Akaike’s information criterion (AIC) was calculated for the models generated. The multivariable model for each outcome was simplified by sequential backward regression, removing the variable with the highest p-value until either 1) all predictors were significantly associated with the outcome, or 2) the best AIC was achieved. Principal component analysis of the covariance matrix was used to reduce dimensionality of the cytokines and examine the primary inflammatory components. Only components with an eigenvalue greater than 1 were then tested for their association with outcomes.

Mediation analysis was conducted to determine to what degree the effect of WFNS grade and blood volume on CT scan on outcome was mediated by CSF and plasma cytokines. The models built had the independent variable as either WFNS grade or blood volume, the principal components or cytokines as mediator variables, and one of the outcome variables listed above. Bootstrapping was used as per Hayes’ PROCESS macro to obtain the standard errors (and p-values) for the indirect effect. Where this failed to yield significant results, Zhao, Lynch & Chen's approach to testing mediation was used and the Monte Carlo test was applied [35]. P < 0.05 was deemed to be significant. All analyses were performed in STATA/IC 18.0.

## Results

A total of 96 patients with SAH recruited between April 2016 and February 2019 were eligible for day 7 cytokine analysis, along with 18 control patients without SAH. Table 1 details the baseline characteristics of all participants. There were no differences in baseline characteristics between patients who had CSF samples collected and patients who did not have their CSF collected. Supplementary Table 1 outlines the baseline characteristics of poor-grade SAH patients (WFNS IV-V) and good-grade SAH patients (WFNS I-III). Supplementary Table 2 describes the baseline characteristics of SAH patients by method of CSF collection (EVD or LP). Compared to patients that had CSF obtained via LP, patients whose CSF was obtained via EVD were of poorer grade, were more likely to have a vertebrobasilar aneurysm, an intraventricular haemorrhage, and a greater blood clot volume on their admission CT.

**Table 1:** Baseline characteristics of subarachnoid haemorrhage (SAH) patients and controls. ^a^ Fisher’s exact. ^b^ Kruskal Wallis. World Federation of Neurosurgical Societies = WFNS; external ventricular drain = EVD

		SAH patients (N = 96)	Control (N = 18)	P-value
Age [median, IQR]		55 (49 – 62.5)	68 (24 – 75)	0.325 ^b^
Sex (n, %)	Female	72 (75.0)	15 (83.3)	0.334 ^a^
	Male	24 (25.0)	3 (16.7)	
Race (n, %)	White	93 (96.9)	NA	NA
	Black	1 (1.0)	NA	
	Asian	2 (2.1)	NA	
Premorbid hypertension	Yes	28 (29.2)	2 (11.1)	0.238 ^a^
	No	68 (70.8)	11 (61.1)	
	Missing	0 (0.0)	5 (27.8)	NA
WFNS Grade (n, %)	I	43 (44.8)	NA	NA
	II	16 (16.7)	NA	
	III	7 (7.3)	NA	
	IV	25 (26.0)	NA	
	V	5 (5.2)	NA	
Blood volume (n = 94) (cm^3^) [median, IQR]		20.1 (8.8 – 34.6)	NA	NA
Intracerebral haemorrhage present (n, %)	Yes	15 (15.6)	NA	NA
	No	80 (83.3)	NA	
	Missing	1 (1.0)	NA	NA
Intraventricular haemorrhage present (n, %)	Yes	71 (74.0)	NA	NA
	No	24 (25.0)	NA	
	Missing	1 (1.0)	NA	NA
EVD inserted (n, %)	Yes	32 (33.3)	NA	NA
	No	64 (66.7)	NA	
Given SFX-01 (n, %)	Yes	45 (46.9)	NA	NA
	No	51 (53.1)	NA	
Aneurysm location (n, %)	Anterior Cerebral	39 (40.6)	NA	NA
	Internal Carotid	17 (17.7)	NA	
	Middle Cerebral	26 (27.1)	NA	
	Vertebrobasilar	12 (12.5)	NA	
	Non-aneurysmal	2 (2.1)	NA	
Securing of the aneurysm (n, %)	Clipping	23 (24.0)	NA	NA
	Coiling	71 (74.0)	NA	
	Not applicable	2 (2.1)	NA	

### Comparison of SAH vs Controls

**Fig. 2:**
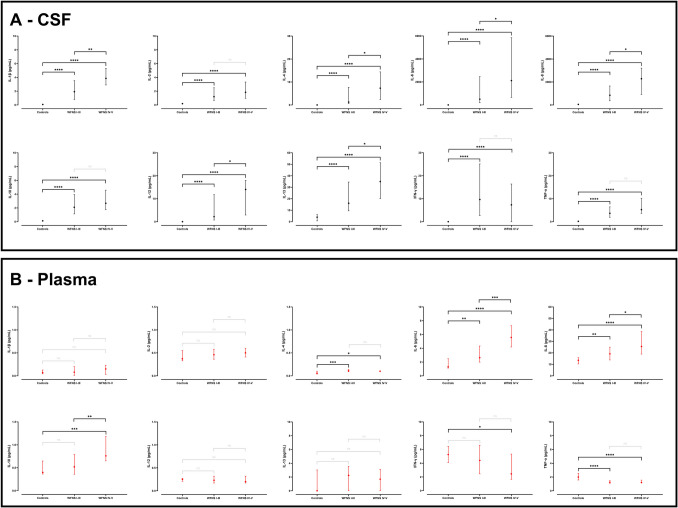
Cytokine levels in the cerebrospinal fluid (CSF) [black vertical lines] and plasma [red vertical lines] of patients with subarachnoid haemorrhage (separated by WFNS grade) and controls. Median & interquartile range shown. Groups compared with Kruskal–Wallis + Dunn’s post hoc test with Bonferroni adjustment. * < 0.05; ** < 0.01; *** < 0.001; **** < 0.0001

**Fig. 3:**
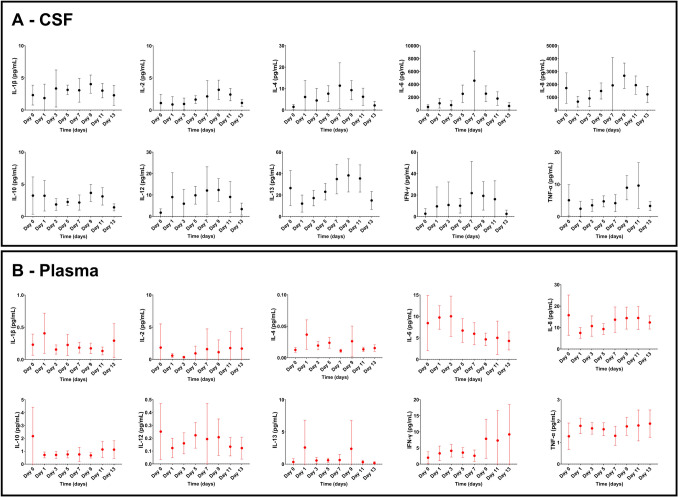
Means (error bars represent 95% confidence interval) of cytokine levels in the (A) cerebrospinal fluid (CSF) and (B) plasma by days after subarachnoid haemorrhage (SAH). Day 0 represents the day of ictus (onset of subarachnoid haemorrhage). The number of patients at each time point is as follows: N = 8 (Day 0), 17 (Day 1), 16 (Day 3), 24 (Day 5), 8 (Day 7), 19 (Day 9), 15 (Day 11), and 13 (Day 13). Data are from patients who had more than one CSF sample taken

On day 7 all CSF cytokines showed marked increases in both good grade and poor grade SAH patients compared to controls (Fig. 2). IL-6 showed markedly larger rises than any other cytokine. This was followed by IL-8, and both showed peak levels two orders of magnitude larger than the rest (Fig. 3). All CSF cytokines remained higher in SAH patients compared to controls when comparing only LP data (Supplementary Table 3).

In plasma, only IL-4, IL-6 and IL-8 were increased in both good grade and poor grade SAH patients compared to controls (and IL-10 if good grade and poor grade SAH patients were considered together), and TNF-α was decreased (and IFN-γ if only poor grade SAH patients were considered). The most marked increase was in plasma IL-6, but the size of this increase was at least two orders of magnitude smaller than CSF IL-6. Similar results were obtained when comparing only LP data (Supplementary Table 3).

### Time Course

The time course of each CSF and plasma cytokine is shown in Fig. 3.

All CSF cytokines displayed visual peaks between days 7 and 11 after symptom onset (day 7 – IL-4, IL-6, and IFN-γ; day 9 – IL-1β, IL-2, IL-8, IL-10, IL-12, and IL-13; day 11 -TNF-α). Amongst the plasma cytokines, only IL-10 and IL-6 displayed clear visual peaks, which occurred earlier than in CSF, at 0 days and 3 days after ictus respectively. Supplementary Table 4 outlines the full results of the within-subjects ANOVA and Tukey’s pairwise comparisons.

### Influence of Surgical Procedures and Interventions

All further analyses were performed on samples from day 7 (± 1 day). Plasma IL-6 and IL-8 levels, and CSF IL-1β, IL-4 and IL-12 levels were significantly greater among patients who had an EVD in situ (Supplementary Table 3). After adjusting for the effect of CT blood volume and/or WFNS, the presence of an EVD did not have any significant effect on any CSF or plasma cytokine levels. There were no differences in CSF or plasma cytokines in patients who underwent coiling and those who underwent clipping (the only difference in plasma IL-12 did not survive Bonferroni correction). There was also no difference in either CSF or plasma cytokines between the SFX-01 and placebo groups.

### Plasma and CSF Cytokine Correlations

**Fig. 4:**
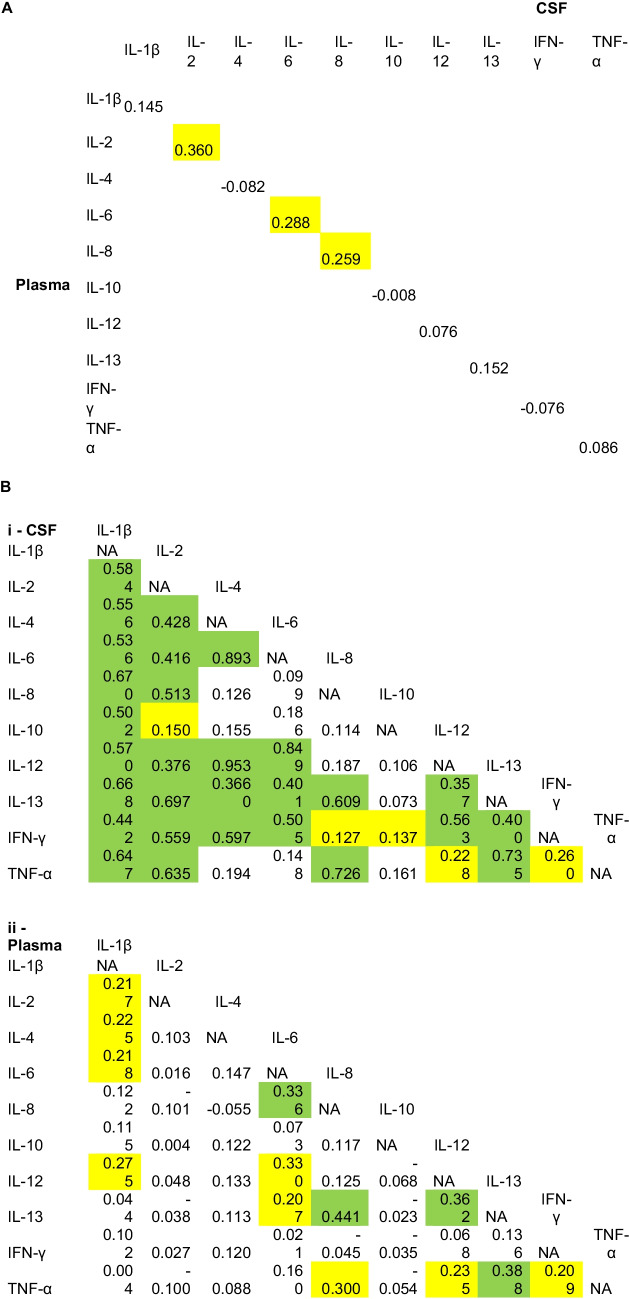
A) Correlation between cytokine levels in the CSF and plasma. B) Correlation between cytokine levels in the i) CSF and ii) plasma. Green represents significant correlations after Bonferroni correction. Yellow represents significant correlations before Bonferroni correction

In patients with SAH, CSF cytokine concentrations were much higher than those in the plasma for all cytokines (Supplementary Table 4). In some cases (such as IL-6 and IL-8) the difference between compartments was striking, reaching several orders of magnitude. There was no significant correlation between CSF and plasma levels for any cytokine (significance for IL-2, IL-6 and IL-8 disappeared on Bonferroni correction) (Fig. 4a).

Within the CSF, seven cytokines correlated with all other CSF cytokines. Additionally, IL-8 correlated with IL-1β, IL-2, IL-13, and TNF- α; IL-10 correlated with IL-1β; and TNF- α correlated with IL-1β, IL-2, IL-8, and IL-13 (Fig. 4b shows correlations before and after Bonferroni correction).

Within the plasma, there was much less correlation between cytokines. There was correlation between IL-6 and IL-8, IL-8 and IL-13, IL-12 and IL-13, and IL-13 and TNF-α (Fig. 4b shows correlations before and after Bonferroni correction).

Since a possible source of plasma cytokines was the CSF compartment, we studied the relationship of plasma and CSF cytokines with qAlb (Supplementary Table 5). There was no evidence that qAlb was predicting plasma cytokines or affecting the relationship between CSF and plasma cytokines.

### Relationship with Clinical Severity

Poor grade SAH patients had significantly higher CSF levels for the majority of cytokines (IL-1β, IL-4, IL-6, IL-8, IL-12, IL-13) compared to good grade SAH patients (Fig. 2) (as well as IL-10 and TNF-α before Bonferroni correction). Only plasma IL-6, IL-8, and IL-10 were significantly elevated in poor grade SAH patients compared to good grade SAH patients (and IFN-γ was significantly decreased before Bonferroni correction).

### Relationship with Blood Volume

CSF cytokines – IL-1β (β: 2.748 [95% CI: 1.131 – 4.363), IL-2 (β: 2.749 [95% CI: 0.917 – 4.580), IL-8 (β: 0.004 [95% CI: 0.002 – 0.006), and TNF-α (β: 1.124 [95% CI: 0.402 – 1.846) – were associated with blood volume (as well as IL-4, IL-6, IL-12, and IL-13 before Bonferroni correction). No plasma cytokines were significantly associated with blood volume on CT (and only IL-8 before Bonferroni correction).

### Vasospasm and DCI

No CSF cytokines were significantly associated with TCD readings at day 7 or DCI in univariable analysis. The absence of a significant association between individual CSF cytokines and DCI persisted in a multivariable regression incorporating all CSF cytokines, and in multivariable regression adjusting for blood volume or WFNS grade.

Plasma IL-1β (β: 88.838 [95% CI: 10.175 – 167.502; p = 0.027]), IL-6 (β: 1.594 [95% CI: 0.034 – 3.155; p = 0.045]), and IL-8 (β: 0.929 [95% CI: 0.266 – 1.592; p = 0.007]) were significantly associated with increased TCD readings at day 7. Plasma IL-6 (OR 1.149 [95% CI: 1.025 – 1.289; p = 0.017]) and IL-8 (OR 1.047 [95% CI: 1.012 – 1.084; p = 0.009]) were significantly associated with DCI.

In a multivariable regression model incorporating plasma cytokines whose levels differed between SAH patients and controls on any comparison (IL-4, IL-6, IL-8, IL-10, IFN-γ and TNF-α), only plasma IL-6 (OR 1.151 [95% CI: 1.021 – 1.297; p = 0.021]) and IL-8 (OR 1.051 [95% CI: 1.009 – 1.096; p = 0.017]) were associated with DCI. Adjusting for either blood volume or WFNS grade, both IL-6 and IL-8 remained significant; but adjusting for both CT blood volume and WFNS grade resulted in no cytokines being associated with DCI.

### Functional Outcome – Univariable Analysis

**Fig. 5:**
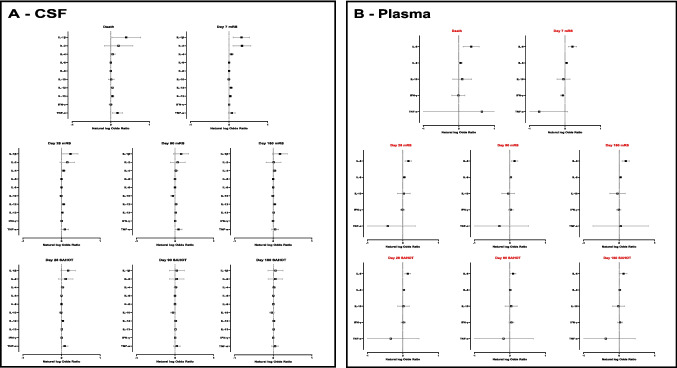
Association of cytokines in the (A) cerebrospinal fluid (CSF) and (B) plasma of patients with subarachnoid haemorrhage with death, delayed cerebral ischaemia (DCI), modified Rankin Scale (mRS), and SAH Outcome Tool (SAHOT) on univariable analysis. Only cytokines that were significantly different between SAH patients and controls are presented. Plasma IL-4 was different in SAH cases compared to controls, but its relationship with outcome is not presented here due to confidence intervals exceeding the scale of the x axis; it was not found to be significant for any outcome measures. White > 0.1; 0.1 > Grey > 0.05; Black < 0.05

Figure 5 presents the results of univariable regression analyses for the associations between outcomes and cytokines that had significantly different levels between SAH patients and controls on any comparison. The full details of all regressions are presented in Supplementary Table 6.

*CSF:* In univariable analysis, no cytokine was associated with all outcome measures (death, mRS and SAHOT) across all time points. Multiple CSF cytokines (IL-1β, IL-2, IL-4, and IL-13) were significantly associated with poorer outcome in the short-term, but not the long-term. Only CSF IL-6 and IL-12 were associated with poorer outcomes on the mRS score at 180 days post-ictus.

*Plasma:* In univariable analysis, the only cytokine significantly associated with all outcome measures (death, mRS and SAHOT) across all timepoints was IL-6. Plasma IL-8 was associated with increased odds of death, and poorer mRS (but not SAHOT) across most timepoints (except day 90) with smaller odds ratios than plasma IL-6. Otherwise, there were no consistent associations of any cytokine across multiple timepoints.

### Functional Outcome – Multivariable Analysis

Supplementary Fig. 1 presents the results of multivariable analyses of all cytokines within the same compartment that were p < 0.1 on univariable analysis for their association with each outcome. The only significant association that remained for CSF cytokines was at day 7 between CSF IL-8 levels and mRS (OR 1.001 [95% CI: 1.000 – 1.001; p = 0.011]). Of the plasma cytokines, IL-6 remained significantly associated with all outcome measures across all timepoints. Additional sensitivity analyses of different regression models are presented in Appendix S1; all showed IL-6 remained significantly associated with all outcome measures across all timepoints.

Further multivariable models were built that assessed the association between each outcome and cytokines that were p < 0.1 on univariable analysis adjusting for CT blood volume and WFNS grade. Only plasma IL-6 was associated with all outcomes across all timepoints (Table 2). The only other significant association was between CSF IL-4 and day 7 mRS.

**Table 2:** Association of cytokine levels in cerebrospinal fluid (CSF) and plasma with outcome variables after adjusting for the effects of cytokines within the same compartment that were p < 0.1 on univariable analysis, World Federation of Neurosurgical Societies (WFNS) and CT blood volume, and then simplifying the model to achieve the best Akaike’s information criterion (AIC). P > 0.10 in light grey, 0.10 > p > 0.05 in dark grey, p < 0.05 in black bold

Multivariable regression			Death	DCI	mRS				SAHOT		
					**Day 7**	**Day 28**	**Day 90**	**Day 180**	**Day 28**	**Day 90**	**Day 180**
CSF	IL-1 β	OR	4.00 0.461 – 34.800	-	-	-	-	-	-	-	-
		*P-value*	*0.209*	-	-	-	-	-	-	-	-
	IL-4	OR	-	0.944 0.861–1.034	**1.287** **1.030–1.606**	-	-	-	-	-	-
		*P-value*	-	*0.211*	***0.026***	-	-	-	-	-	-
	IL-6	OR	-	-	1.000 0.999–1.000	-	-	1.000 1.000–1.000	-	1.00 1.000–1.000	-
		*P-value*	-	-	*0.080*	-	-	*0.408*	-	*0.402*	-
	IL-10	OR	-	-	-	-	0.934 0.867–1.008	-	-	-	-
		*P-value*	-	-	-	-	*0.078*	-	-	-	-
	IL-12	OR	-	-	0.890 0.781–1.015	1.026 0.989–1.064	-	-	-	-	-
		*P-value*	-	-	*0.083*	*0.166*	-	-	-	-	-
	TNF-α	OR	-	-	-	-	-	-	1.024 0.943–1.112	-	-
		*P-value*	-	-	-	-	-	-	*0.577*	-	-
Plasma	IL-6	OR	4.635 0.221–97.184	**1.163** **1.015 – 1.334**	**1.167** **1.037 – 1.314**	**1.111** **1.030 – 1.198**	**1.127** **1.026 – 1.238**	**1.174** **1.060 – 1.300**	**1.133** **1.038 – 1.235**	1.078 0.994 – 1.169	**1.118** **1.015 – 1.231**
		P-value	*0.323*	***0.030***	***0.010***	***0.007***	***0.012***	***0.002***	***0.005***	*0.069*	***0.023***
	IL-8	OR	0.821 0.521 – 1.295	1.039 0.995 – 1.085	-	-	-	-	-	-	-
		P-value	*0.396*	*0.080*	-	-	-	-	-	-	-
	IFN-γ	OR	-	0.666 0.433 – 1.026	-	-	-	-	-	-	-
		P-value	-	*0.065*	-	-	-	-	-	-	-
	Blood Volume	OR	1.293 0.683 – 2.448	**1.054** **1.009 – 1.101**	**1.044** **1.009 – 1.081**	**1.037** **1.008 – 1.068**	**1.037** **1.011 – 1.064**	**1.032** **1.006 – 1.059**	**1.042** **1.015 – 1.070**	**1.038** **1.010 – 1.066**	**1.036** **1.012 – 1.060**
		P-value	*0.430*	***0.018***	***0.015***	***0.014***	***0.005***	***0.015***	***0.002***	***0.008***	***0.003***
	WFNS	OR	-	-	**25.068** **6.200 – 101.353**	**3.661** **1.325 – 10.116**	-	-	-	-	-
		P-value	-	-	** < *0.001***	***0.012***	-	-	-	-	-

OR = Odds Ratio expressed as coefficient (95% CI).

- indicates cytokine did not meet the threshold for inclusion in the multivariable analysis (p < 0.1 in univariable analysis).

### Principal Component Analysis

In order to reduce dimensionality of the cytokine data and reduce the risk of false negatives due to multicollinearity, principal component analysis (on the covariance matrix) was undertaken (Table 3).

**Table 3:** Association of principal components of cytokine levels in cerebrospinal fluid (CSF) and plasma with outcome variables after adjusting for the effects of all other principal components that were p < 0.1 on univariable analysis, World Federation of Neurosurgical Societies (WFNS) and CT blood volume, and then simplifying the model to achieve the best Akaike’s information criterion (AIC). P > 0.10 in light grey, 0.10 > p > 0.05 in dark grey, p < 0.05 in black bold

Multivariable regression			Death (OR)	DCI (OR)	MRS (OR)				SAHOT (OR)		
					**Day 7**	**Day 28**	**Day 90**	**Day 180**	**Day 28**	**Day 90**	**Day 180**
CSF	Principal Component 1	OR	1.759 0.975 – 3.175	-	1.144 0.920 – 1.423	1.089 0.898 – 1.320	-	-	1.062 0.887 – 1.271	-	-
		*P-value*	*0.061*	*-*	*0.227*	*0.387*	*-*	*-*	*0.513*	-	-
	Principal Component 3	OR	-	-	-	-	0.600 0.346 – 1.040	-	-	-	-
		*P-value*	-	-	-	-	*0.069*	*-*	-	-	-
Plasma	Principal Component 1	OR	**3.207** **1.166 – 8.819**	1.331 0.874 – 2.028	-	1.309 0.968 – 1.769	1.341 1.000 – 1.798	**1.525** **1.156 – 2.012**	-	1.311 0.998 – 1.721	**1.440** **1.054 – 1.968**
		*P-value*	***0.024***	*0.183*	*-*	*0.080*	*0.050*	***0.003***	*-*	*0.052*	***0.022***
	Principal Component 2	OR	-	-	-	-	-	-	-	**1.425** **1.023 – 1.984**	-
		*P-value*	-	-	-	-	-	-	-	***0.036***	-
	Principal Component 3	OR	-	0.560 0.311 – 1.009	-	-	-	-	-	-	-
		*P-value*	-	*0.053*	*-*	-	-	-	-	-	-
	Principal Component 4	OR	-	-	**0.581** **0.360 – 0.936**	-	-	0.734 (0.513 – 1.048)	-	-	-
		*P-value*	-	-	***0.026***	*-*	*-*	*0.089*	-	-	-

OR = Odds Ratio expressed as coefficient (95% CI).

- indicates principal component did not meet the threshold for inclusion in the multivariable analysis (p < 0.1 in univariable analysis).

Principal component analysis of CSF cytokines identified three principal components with eigenvalues greater than 1.0. The scree plot in Supplementary Fig. 2a shows that the first three principal components explained most of the variance in the data. Supplementary Fig. 2b shows the eigenvectors of the cytokines that form each CSF principal component. Four principal components with an eigenvalue greater than 1.0 were identified from the plasma cytokines. The scree plot in Supplementary Fig. 3a shows that the first four principal components explained most of the variance in the data. Supplementary Fig. 3b shows the eigenvectors of the cytokines that form each plasma principal component.

Supplementary Table 7 presents the results of the univariable regression analyses for the associations between outcomes and principal components. The first CSF principal component was the only CSF principal component significantly associated with death and worse mRS outcomes (not SAHOT outcomes), but this was only for short-term outcomes.

On backward multivariable regression considering all plasma principal components that were p < 0.1 on univariable analysis for their association with each outcome, the first plasma principal component (dominated by IL-6, IL-8, IL-12, IL-13, and TNF-α, i.e., their principal component coefficients were larger than the equilibrium contribution 1/√10 = 0.316 (3 d.p.)) was the only plasma principal component associated with worse mRS and SAHOT at days 90 and 180 (Supplementary Fig. 4). Additional sensitivity analyses of different regression models are presented in Appendix S2, which all showed the first plasma principal component remained significantly associated with long-term outcome measures.

Further multivariable models were built that assessed the association between each outcome and principal components that were p < 0.1 on univariable analysis adjusting for CT blood volume and WFNS grade. Only plasma principal component 1 was associated with long term outcome (mRS and SAHOT at day 180) (Table 3).

### Mediation analysis

Each of the cytokines and PCs with a p < 0.1 on univariable analysis (Supplementary Table 7) was individually tested on mediation analysis to see if they mediated any of the effects of WFNS or blood volume on outcome. Supplementary Fig. 5 gives an example of the models tested. Bootstrapping found that none of the indirect effects tested were significant, and therefore none of the effect of WFNS grade and blood volume on outcome was mediated by any cytokines or their principal components (including plasma IL-6 and plasma PC1).

Using the Monte Carlo test, there was only marginal evidence for plasma IL-6 mediation of the effect of WFNS grade on mRS at 28 days post-ictus (p = 0.049) with only 0.124 of the total effect of WFNS grade mediated by plasma IL-6 and no mediation seen at any other timepoint. No other cytokines were found to mediate an effect of WFNS or blood volume on long-term outcomes.

## Discussion

### Key Findings

In this study, we have shown that the inflammatory responses after SAH in the CSF and plasma compartments are distinct and independent of each other. While there was a tight correlation between different cytokines within the CSF, and some correlation between cytokines within the plasma, the timing and magnitude of the responses in the CSF and plasma were markedly different, with the CSF response being delayed (day 7–9 vs day 0–3) and much higher (by 1–3 orders of magnitude), compared to plasma. Consistent with the notion of two independent compartmentalised cytokine responses, there was no correlation between CSF and plasma compartments, even when considering an interaction with Qalb.

The main drivers of the CSF inflammatory response were the size of the bleed and the neurological condition of the patient on admission as evidenced by the independent association of the CSF cytokines with blood volume on CT and WFNS grade. The delayed timing of the CSF response relative to the plasma response, and its independent nature, suggests that this is driven by secondary processes within the cranial compartment. The association with blood volume suggests a possible linkage to blood products released from the blood clot. The much earlier plasma response was seen predominantly in IL-6 and was not correlated with bleed severity. This suggests it is driven more by the early injury, and its lack of correlation with bleed severity reflects a different systemic response to that in the CNS.

Although many cytokine-outcome combinations were tested, only plasma IL-6 (and to a lesser extent plasma IL-8) was consistently associated with outcome. The strongest associations were with plasma, not CSF IL-6. Interestingly this applied to vasospasm and DCI as well as long term functional outcome. Principal component analysis identified only the first plasma component was associated with long-term mRS and SAHOT assessments. However, this component’s predictive value was less than that of plasma IL-6 alone, suggesting the effect of this component was driven by IL6, and the contribution from other cytokines detracted from this. In terms of mechanism, mediation analysis showed that the effect of plasma IL-6 on outcome was not by mediating the effect of WFNS or blood volume on long-term outcome.

### Results in context of previous studies

There is a considerable existing literature describing inflammation after SAH. Our study is comparatively large in terms of the number of paired blood and CSF samples available, and the number of cytokines considered, and unusual due to the availability of CSF from patients without EVDs. This has made comparison of good and poor outcome patients possible and allowed us for the first time to make meaningful comparisons between the systemic and CNS inflammatory response. Supplementary Table 8 details the previously published literature and appendix S3 summarises what was previously known and what is new, and discusses the similarities and differences to contextualise our findings.

In summary, thirteen studies have measured IL-6 in both CSF and plasma/serum, and have reported significantly higher levels of IL-6 in the CSF [15, 17, 36–46]. Additionally, several studies have reported on the association between CSF IL-6 and outcomes [19, 22, 28, 47–53]. These studies reported conflicting results. The discrepancies may arise from the timing of CSF sampling or timing of outcome measurement. As seen in our study, CSF cytokines may be associated with outcome in the short-term, but not in the long-term. Furthermore, the studies that found an association between CSF IL-6 levels and unfavourable outcomes in the long-term only did so on univariable analysis [28, 36, 37, 43, 54]. Given CSF cytokines are strongly associated with blood volume and WFNS grade, it is important to correct for these baseline variables.

Previous studies have produced findings which differed in some aspects from ours, and this may be potentially explained by differences in study design. The first study was a small series (n = 24) which found that levels of cytokines were highest in the plasma in the first few days after SAH – similar to our study – but CSF cytokines peaked at ictus and around day 7 [42], though steroid treatment may have altered the temporal pattern of CSF cytokines. The second study found that IL-6 and IL-8 levels in the CSF were significantly greater within 3 days after SAH, compared to day 10 [55], but the samples from day 3 and 10 were obtained from two different populations. With a larger sample size, paired CSF/serum sampling and a panel of ten cytokines in the same cohort, we provide definitive evidence that the temporal behaviour of cytokine levels is distinctly different in plasma and CSF.

Another study found higher levels of CSF IL-6 within 72 h from ictus was associated with vasospasm [55]. In contrast, we did not find a significant association between CSF IL-6 levels at day 7 and TCDs. Taken together with our study, these findings could be interpreted as demonstrating that CSF IL-6 levels at an earlier stage predict vasospasm, but no longer do so at day 7. This is supported by another study, which found CSF IL-6 on day 6 and 7 was not associated with vasospasm, but CSF from days 2–4 was (day 4 having the strongest association with vasospasm) [19]. Therefore, it may be beneficial to antagonise the effects of CSF IL-6 acutely during early brain injury to prevent vasospasm and improve outcomes.

### Implications

Our results show that CSF and plasma inflammatory responses are distinct and separate. The lower levels of cytokines in the plasma argue against a plasma origin for CSF cytokines. The much higher concentrations of cytokines in the CSF raise the theoretical possibility of brain-to-blood efflux of cytokines, but the lack of correlation between plasma and CSF cytokines, and the lack of an interaction with qAlb makes this unlikely. Furthermore, computational analyses suggest that cytokines typically traverse only a few cell diameters before being consumed [56, 57]. Finally there is experimental evidence that cytokines can be produced by cells found within the central nervous system [58] after SAH. Taken together this suggests that the production of cytokines occurs discretely in each compartment, and that cytokines do not cross the BBI in significant quantities after SAH. However, the levels of cytokines were measured at day 7, and it could be possible that there is movement across the BBI at earlier time points.

This has several important implications. First, it suggests that plasma and CSF cytokines are poor surrogates for each other. This means that future studies need to sample from the compartment of interest depending on the hypothesis being tested. Second, our data suggests that CSF cytokines are increased as a response to the bleed, but the effect of this is not deleterious in itself (or any deleterious effect is sufficiently small not to be detectable in a study this size). Therefore, CSF cytokines levels at day 7 may be a poor marker for prognosis from delayed brain injury and may not serve as a therapeutic target to improve long-term outcomes if treatments targeting cytokines are started after the acute period.

Most interesting is the observation that plasma IL-6 levels appear to be independently associated with outcome, and not by mediating the effects of WFNS and blood volume on outcome. The pathway by which plasma IL-6 influences outcome is not clear. Although, plasma IL-6 was associated with vasospasm and DCI and its peak preceded their expected onset, these complications only make modest contributions to outcome [59] including in this dataset, and they did not mediate the effect of plasma IL-6 on outcome (data not shown). The IL-1 receptor antagonist (IL-1RA) Kineret—currently being tested in a Phase 3 randomised controlled trial [60]– antagonises IL-1β and prevents induction of IL-6 in both CSF and plasma [61, 62]. Given that our data shows that plasma but not CSF IL-6 is associated with outcome, it is possible that central nervous system penetration is not essential for IL1-RA’s mechanism of action if it is proven to be an efficacious treatment.

### Limitations

Several limitations should be acknowledged. Although much larger than any previous study of paired plasma and CSF, the sample size was still modest, and would therefore not be powered to detect small differences. Furthermore, the statistical power is limited by multiple testing. The primary analyses were limited to day 7, which may not be representative of other timepoints. The day 7 sampling was selected to align with the peak of Hb exposure and vasospasm and coincides with the CSF cytokine peak originally anticipated to be linked to outcome. However, the study has unexpectedly shown that it is the plasma not CSF cytokine profile which is clinically relevant. We focused on a limited set of cytokines and did not investigate the potential roles of other inflammatory mediators. The cytokine assay did not distinguish between inactive or active cytokine forms. Half the SAH patients in this study received SFX-01 which contains sulforaphane which may have influenced our findings. However, a sensitivity analysis showed there was no difference in inflammatory markers between SAH patients who received SFX-01 or placebo and neither exclusion of treated patients nor controlling for SFX-01 use altered any of our findings. The CSF samples were collected via an EVD or LP, and this may be seen to introduce heterogeneity due to the rostro-caudal gradient in CSF protein content [63, 64]. However, after adjusting for the effect of CT blood volume and/or WFNS, the presence of an EVD did not have any significant effect on any CSF or plasma cytokine levels.

## Conclusion

This study shows that the inflammatory response in the CSF and plasma compartments are distinct and different, suggesting separate roles in SAH pathology. In the CSF there is a strong pan-cytokine response whereas in plasma it is focussed on IL-6. Surprisingly it is the plasma IL-6 rather than the CSF cytokine response that is associated with outcome. Further research is needed to understand the mechanisms underlying the association of plasma IL-6 with outcome.

## Supplementary Information

Below is the link to the electronic supplementary material.

Supplementary file1 (DOCX 485 KB)

## Data Availability

The datasets used and/or analysed during the current study are available from the corresponding author on reasonable request.
