# Supplementary material for: Distinct Cytokine Responses in Central and Systemic Compartments after Subarachnoid Haemorrhage
Source: Transl Stroke Res. 2025 Mar 25;16(5):1766–82. doi: 10.1007/s12975-025-01348-y (PMC12391180; doi:10.1007/s12975-025-01348-y)
Supplement: Supplementary file 1 — Supplementary file1 (DOCX 485 KB) [file 12975_2025_1348_MOESM1_ESM.docx]

**Supplementary Table 1 –** Baseline characteristics by WFNS grade of subarachnoid haemorrhage patients. ^a^ Fisher’s exact. ^b^ Kruskal Wallis. World Federation of Neurosurgical Societies = WFNS; external ventricular drain = EVD.

|  | | WFNS grade  I-III (N = 66) | WFNS grade  IV-V (N = 30) | P-value |
| --- | --- | --- | --- | --- |
| Age  [median, IQR] | | 55  (49 – 62) | 55.5  (50 – 65) | 0.610 ^b^ |
| Sex (n, %) | Female | 46 (69.7) | 26 (86.7) | 0.083 ^a^ |
|  | Male | 20 (30.3) | 4 (13.3) |  |
| Race (n, %) | White | 64 (97.0) | 29 (96.7) | 0.379 ^a^ |
|  | Black | 0 (0.0) | 1 (3.3) |  |
|  | Asian | 2 (3.0) | 0 (0.0) |  |
| Premorbid hypertension | Yes | 21 (31.8) | 7 (23.3) | 0.473 ^a^ |
|  | No | 45 (68.2) | 23 (76.7) |  |
| WFNS Grade (n, %) | I | 43 (65.2) | NA | NA |
|  | II | 16 (24.2) | NA |  |
|  | III | 7 (10.6) | NA |  |
|  | IV | NA | 25 (83.3) |  |
|  | V | NA | 5 (16.7) |  |
| Blood volume (cm^3^) [median, IQR] | | 15.9  (7.1 – 32.4) | 30.9  (16.9 – 47.5) | < 0.001 ^b^ |
| Intracerebral haemorrhage present (n, %) | Yes | 8 (12.1) | 7 (23.3) | 0.227 ^a^ |
|  | No | 57 (86.4) | 23 (76.7) |  |
|  | Missing | 1 (1.5) | 0 (0.0) | NA |
| Intraventricular haemorrhage present (n, %) | Yes | 43 (65.2) | 28 (93.3) | 0.005 ^a^ |
|  | No | 22 (33.3) | 2 (6.7) |  |
|  | Missing | 1 (1.5) | 0 (0.0) | NA |
| EVD inserted (n, %) | Yes | 11 (16.7) | 21 (70.0) | < 0.001 ^a^ |
|  | No | 55 (83.3) | 9 (30.0) |  |
| Given SFX-01 (n, %) | Yes | 33 (50.0) | 12 (40.0) | 0.387 ^a^ |
|  | No | 33 (50.0) | 18 (60.0) |  |
| Aneurysm location (n, %) | Anterior Cerebral | 30 (45.4) | 9 (30.0) | 0.057 ^a^ |
|  | Internal Carotid | 13 (19.7) | 4 (13.3) |  |
|  | Middle Cerebral | 17 (25.8) | 9 (30.0) |  |
|  | Vertebrobasilar | 4 (6.1) | 8 (26.7) |  |
|  | Non-aneurysmal | 2 (3.0) | 0 (0.0) |  |
| Securing of the aneurysm (n, %) | Clipping | 16 (24.2) | 7 (23.3) | 1.000 ^a^ |
|  | Coiling | 48 (72.7) | 23 (76.7) |  |
|  | Not applicable | 2 (3.0) | 0 (0.0) |  |

**Supplementary Table 2 –** Baseline characteristics by method of CSF collection for subarachnoid haemorrhage patients. ^a^ Fisher’s exact. ^b^ Kruskal Wallis. World Federation of Neurosurgical Societies = WFNS; lumbar puncture = LP; external ventricular drain = EVD.

|  | | LP (N = 51) | EVD (N = 32) | P-value |
| --- | --- | --- | --- | --- |
| Age  [median, IQR] | | 55  (50 – 62) | 56  (49.5 – 66.5) | 0.694 ^b^ |
| Sex (n, %) | Female | 38 (74.5) | 26 (81.3) | 0.333 ^a^ |
|  | Male | 13 (25.5) | 6 (18.8) |  |
| Race (n, %) | White | 49 (96.1) | 32 (100.0) | 0.520 ^a^ |
|  | Black | 0 (0.0) | 0 (0.0) |  |
|  | Asian | 2 (3.2) | 0 (0.0) |  |
| Premorbid hypertension | Yes | 16 (31.4) | 7 (21.9) | 0.452 ^a^ |
|  | No | 35 (68.6) | 25 (78.1) |  |
| WFNS Grade (n, %) | I | 32 (62.8) | 3 (9.4) | < 0.001 ^a^ |
|  | II | 9 (17.7) | 5 (15.6) |  |
|  | III | 4 (7.8) | 3 (9.4) |  |
|  | IV | 6 (11.8) | 17 (53.1) |  |
|  | V | 0 (0.0) | 4 (12.5) |  |
| Blood volume (cm^3^) [median, IQR] | | 17.3  (7.6 – 31.2) | 28.5  (11.9 – 44.9) | 0.022 ^b^ |
| Intracerebral haemorrhage present (n, %) | Yes | 7 (13.7) | 5 (15.6) | 1.000 ^a^ |
|  | No | 44 (86.3) | 27 (84.4) |  |
| Intraventricular haemorrhage present (n, %) | Yes | 32 (62.8) | 31 (96.9) | < 0.001 ^a^ |
|  | No | 19 (37.3) | 1 (3.1) |  |
| Given SFX-01 (n, %) | Yes | 27 (52.9) | 11 (34.4) | 0.117 ^a^ |
|  | No | 24 (47.1) | 21 (65.6) |  |
| Aneurysm location (n, %) | Anterior Cerebral | 22 (43.1) | 10 (31.3) | 0.050 ^a^ |
|  | Internal Carotid | 11 (21.6) | 4 (12.5) |  |
|  | Middle Cerebral | 13 (25.5) | 9 (28.1) |  |
|  | Vertebrobasilar | 3 (5.9) | 9 (28.1) |  |
|  | Non-aneurysmal | 2 (3.9) | 0 (0.0) |  |
| Securing of the aneurysm (n, %) | Clipping | 10 (19.6) | 10 (31.3) | 0.351 ^a^ |
|  | Coiling | 39 (76.5) | 22 (68.8) |  |
|  | Not applicable | 2 (3.9) | 0 (0.0) |  |

**Supplementary Table 3** – Cytokine levels in the cerebrospinal fluid (CSF) and plasma of controls and of patients 7 days after subarachnoid haemorrhage (SAH). The Wilcoxon-Signed Rank test was used to compare median cytokine levels in paired plasma and CSF samples from the same patients. The Wilcoxon Rank-Sum test was used to compare median cytokine levels in plasma and CSF samples from patients who had lumbar punctures (LPs) against those who had an external ventricular drain (EVD) in situ.

| **Cytokines** | **Plasma levels in all SAH patients (pg/mL)** | **CSF levels in all SAH patients (pg/mL)** | **P-value** | **Fold Difference (CSF vs Plasma)** | **Plasma levels in SAH patients who had a LP (pg/mL)** | **Plasma levels in SAH patients with an EVD (pg/mL)** | **P-value** | **CSF levels in in SAH patients who had a LP (pg/mL)** | **CSF levels in SAH patients with an EVD (pg/mL)** | **P-value** |
| --- | --- | --- | --- | --- | --- | --- | --- | --- | --- | --- |
| IL-1beta  (median, IQR) | 0.09 (0.01 – 0.22) | 2.74 (1.06 – 4.26) | <0.001 | 30.44 | 0.15 (0.01 – 0.24) | 0.04 (0.01 -0.18) | 0.104 | 2.18 (0.85 – 3.76) | 3.62 (1.4 – 5.03) | 0.046 |
| IL-2  (median, IQR) | 0.48 (0.36 – 0.60) | 1.43 (0.71 – 3.02) | <0.001 | 2.98 | 0.48 (0.37 – 0.64) | 0.5 (037 – 0.55) | 0.557 | 1.43 (0.77 – 2.73) | 1.32 (0.57 – 3.03) | 0.757 |
| IL-4  (median, IQR) | 0.11 (0.09 – 0.13) | 2.46 (0.68 – 10.51) | <0.001 | 22.36 | 0.12 (0.10 – 0.13) | 0.10 (0.09 – 0.11) | 0.091 | 1.44 (0.60 – 7.73) | 6.21 (1.17 – 13.19) | 0.031 |
| IL-6  (median, IQR) | 3.45 (2.04 – 5.81) | 802.74  (256.25 – 3936.11) | <0.001 | 232.68 | 2.79 (2.00 – 4.62) | 4.67 (2.37 – 7.28) | 0.046 | 545.03 (201.69 – 2554.26) | 1751.00 (460.66 – 4693.41) | 0.073 |
| IL-8  (median, IQR) | 21.76 (14.86 – 26.71) | 1013.09  (540.63 – 2531.08) | <0.001 | 46.56 | 18.87 (13.29 – 25.33) | 24.82 (17.23 – 42.39) | 0.024 | 918.49 (511.45 – 2207.90) | 1320.55 (611.74 – 2867.63) | 0.154 |
| IL-10  (median, IQR) | 0.67 (0.43 – 0.90) | 2.45 (1.22 – 4.09) | <0.001 | 3.66 | 0.56 (0.43 – 0.79) | 0.75 (0.50 – 1.18) | 0.067 | 2.45 (1.22 – 4.21) | 2.43 (1.25 – 4.03) | 0.743 |
| IL-12  (median, IQR) | 0.23 (0.17 – 0.32) | 3.04 (0.92 – 15.57) | <0.001 | 13.22 | 0.23 (0.16 – 0.30) | 0.20 (0.17 – 0.32) | 0.827 | 2.23 (0.85 – 12.93) | 12.35 (1.92 – 17.37) | 0.026 |
| IL-13  (median, IQR) | 1.89 (0.01 – 3.54) | 21.66 (11.75 – 41.67) | <0.001 | 11.46 | 1.70 (0.01 – 3.25) | 1.88 (0.01 – 3.81) | 0.325 | 17.69 (9.85 – 35.57) | 27.16 (15.02 – 51.85) | 0.070 |
| IFN-gamma  (median, IQR) | 4.13 (2.13 – 6.61) | 7.98 (1.85 – 20.78) | <0.001 | 1.93 | 4.30 (2.33 – 6.53) | 3.18 (1.31 – 7.29) | 0.244 | 11.02 (2.8 – 26.72) | 6.97 (0.01 – 15.03) | 0.357 |
| TNF-alpha  (median, IQR) | 1.23 (1.04 – 1.50) | 4.20  (2.50 – 7.92) | <0.001 | 3.41 | 1.23 (1.07 – 1.44) | 1.14 (0.98 – 1.41) | 0.249 | 3.75 (2.18 – 7.22) | 4.75 (3.20 – 10.58) | 0.213 |
| **Cytokines (pg/mL)** | **Plasma levels in controls** | **CSF levels in controls** | **P-value** | **Fold Difference (CSF vs Plasma)** | **NA** | **NA** | **NA** | **NA** | **NA** | **NA** |
| IL-1beta  (median, IQR) | 0.07 (0.05 – 0.13) | 0.07 (0.03 – 0.09) | 0.253 | 1.00 | **NA** | **NA** | **NA** | **NA** | **NA** | **NA** |
| IL-2  (median, IQR) | 0.37 (0.33 – 0.55) | 0.19 (0.11 – 0.27) | 0.109 | 0.51 | **NA** | **NA** | **NA** | **NA** | **NA** | **NA** |
| IL-4  (median, IQR) | 0.06 (0.04 – 0.10) | 0.01 (0.01 – 0.04) | 0.002 | 0.17 | **NA** | **NA** | **NA** | **NA** | **NA** | **NA** |
| IL-6  (median, IQR) | 1.30 (1.13 – 2.48) | 2.49 (1.55 – 2.91) | 0.225 | 1.92 | **NA** | **NA** | **NA** | **NA** | **NA** | **NA** |
| IL-8  (median, IQR) | 13.51 (10.40 – 15.82) | 48.37 (41.47 - 60.87) | < 0.001 | 3.58 | **NA** | **NA** | **NA** | **NA** | **NA** | **NA** |
| IL-10  (median, IQR) | 0.40 (0.37 – 0.65) | 0.12 (0.09 – 0.17) | < 0.001 | 0.30 | **NA** | **NA** | **NA** | **NA** | **NA** | **NA** |
| IL-12  (median, IQR) | 0.25 (0.21 – 0.27) | 0.01 (0.01 – 0.01) | 0.002 | 0.04 | **NA** | **NA** | **NA** | **NA** | **NA** | **NA** |
| IL-13  (median, IQR) | 0.01 (0.01 – 3.03) | 3.94 (0.73 - 6.18) | 0.126 | 394 | **NA** | **NA** | **NA** | **NA** | **NA** | **NA** |
| IFN-gamma  (median, IQR) | 5.26 (4.09 – 6.45) | 0.01 (0.01 – 0.01) | < 0.001 | 0.02 | **NA** | **NA** | **NA** | **NA** | **NA** | **NA** |
| TNF-alpha  (median, IQR) | 2.00 (1.57 – 2.56) | 0.16 (0.01 – 0.22) | < 0.001 | 0.08 | **NA** | **NA** | **NA** | **NA** | **NA** | **NA** |

**Supplementary Table 4** – Within-subjects ANOVA and Tukey’s pairwise comparisons of significant differences in cytokine levels over time

| **Cytokine** | **P-value** | **Days that were significantly different** | **Tukey corrected p-value** |
| --- | --- | --- | --- |
| CSF IL-1beta | 0.326 | NA | NA |
| CSF IL-2 | 0.004 | Day 9 vs 0 | 0.029 |
| CSF IL-4 | 0.100 | NA | NA |
| CSF IL-6 | < 0.001 | Day 5 vs 0 | 0.003 |
|  |  | Day 7 vs 0 | 0.004 |
|  |  | Day 9 vs 0 | 0.002 |
|  |  | Day 11 vs 0 | 0.024 |
| CSF IL-8 | < 0.001 | Day 9 vs 1 | 0.002 |
|  |  | Day 11 vs 1 | 0.048 |
|  |  | Day 9 vs 3 | 0.015 |
| CSF IL-10 | 0.169 | NA | NA |
| CSF IL-12 | 0.227 | NA | NA |
| CSF IL-13 | < 0.001 | Day 9 vs 1 | 0.04 |
|  |  | Day 13 vs 9 | 0.033 |
|  |  | Day 13 vs 11 | 0.038 |
| CSF IFN-gamma | 0.335 | NA | NA |
| CSF TNF-alpha | 0.034 | Nil | NA |
| Plasma IL-1beta | 0.083 | NA | NA |
| Plasma IL-2 | 0.120 | NA | NA |
| Plasma IL-4 | 0.600 | NA | NA |
| Plasma IL-6 | < 0.001 | Day 9 vs 1 | 0.039 |
|  |  | Day 9 vs 3 | 0.003 |
|  |  | Day 11 vs 3 | 0.016 |
|  |  | Day 13 vs 3 | 0.037 |
| Plasma IL-8 | 0.005 | Day 11 vs 1 | 0.025 |
| Plasma IL-10 | < 0.001 | Day 1 vs 0 | < 0.001 |
|  |  | Day 3 vs 0 | 0.001 |
|  |  | Day 5 vs 0 | < 0.001 |
|  |  | Day 9 vs 0 | < 0.001 |
|  |  | Day 11 vs 0 | 0.011 |
|  |  | Day 13 vs 0 | 0.014 |
| Plasma IL-12 | 0.911 | NA | NA |
| Plasma IL-13 | 0.521 | NA | NA |
| Plasma IFN-gamma | 0.329 | NA | NA |
| Plasma TNF-alpha | 0.560 | NA | NA |

**Supplementary Table 5 –** Relationship between plasma and CSF cytokines among all patients in a multivariable linear regression testing whether a specific CSF cytokine and qAlb predicted the corresponding plasma cytokine, while also considering a possible interaction between qAlb and the CSF cytokine.

| **Plasma cytokine** | **CSF cytokine and qAlb** | **Without Interaction** | **With Interaction** |
| --- | --- | --- | --- |
| IL-1beta | Cytokine, coefficient | 0.006  (-0.006 – 0.019) | 0.007  (-0.010 – 0.024) |
|  | Cytokine, p-value | 0.318 | 0.439 |
|  | qAlb, coefficient | 0.416  (-0.554 – 1.386) | 0.636  (-6.248 – 7.520) |
|  | qAlb, p-value | 0.396 | 0.855 |
|  | Interaction, coefficient | NA | -0.043  (-1.364 – 1.279) |
|  | Interaction, p-value | NA | 0.949 |
|  | R^2^ | 0.0275 | 0.0275 |
| IL-2 | Cytokine, coefficient | 0.163  (0.074 – 0.251) | 0.148  (0.046 – 0.250) |
|  | Cytokine, p-value | < 0.001 | 0.005 |
|  | qAlb, coefficient | -4.430  (-10.625 – 1.765) | -13.569  (-45.955 – 18.816) |
|  | qAlb, p-value | 0.158 | 0.406 |
|  | Interaction, coefficient | NA | 1.573  (-3.897 – 7.042) |
|  | Interaction, p-value | NA | 0.568 |
|  | R^2^ | 0.1526 | 0.1563 |
| IL-4 | Cytokine, coefficient | -0.000  (-0.001 – 0.001) | -0.000  (-0.002 – 0.002) |
|  | Cytokine, p-value | 0.512 | 0.871 |
|  | qAlb, coefficient | -0.040  (-0.313 – 0.233) | -0.018  (-0.408 – 0.371) |
|  | qAlb, p-value | 0.771 | 0.926 |
|  | Interaction, coefficient | NA | -0.008  (-0.109 – 0.093) |
|  | Interaction, p-value | NA | 0.876 |
|  | R^2^ | 0.0070 | 0.0074 |
| IL-6 | Cytokine, coefficient | 0.001  (0.000 – 0.001) | 0.000  (-0.000 – 0.001) |
|  | Cytokine, p-value | 0.014 | 0.333 |
|  | qAlb, coefficient | 0.762  (-49.450 – 50.973) | -8.067  (-89.296 – 73.163) |
|  | qAlb, p-value | 0.976 | 0.844 |
|  | Interaction, coefficient | NA | 0.006  (-0.039 – 0.051) |
|  | Interaction, p-value | NA | 0.783 |
|  | R^2^ | 0.0785 | 0.0795 |
| IL-8 | Cytokine, coefficient | 0.002  (0.000 – 0.004) | 0.002  (0.000 – 0.005) |
|  | Cytokine, p-value | 0.030 | 0.040 |
|  | qAlb, coefficient | 17.274  (-107.071 – 141.619) | 78.625  (-477.870 – 635.121) |
|  | qAlb, p-value | 0.783 | 0.779 |
|  | Interaction, coefficient | NA | -0.010  (-0.099 – 0.079) |
|  | Interaction, p-value | NA | 0.822 |
|  | R^2^ | 0.0740 | 0.0746 |
| IL-10 | Cytokine, coefficient | -0.002  (-0.056 – 0.052) | 0.052  (-0.326 – 0.431) |
|  | Cytokine, p-value | 0.944 | 0.785 |
|  | qAlb, coefficient | -1.334  (-17.599 – 14.931) | 13.719  (-92.185 – 119.623) |
|  | qAlb, p-value | 0.871 | 0.797 |
|  | Interaction, coefficient | NA | -3.929  (-31.239 – 23.381) |
|  | Interaction, p-value | NA | 0.775 |
|  | R^2^ | 0.0004 | 0.0015 |
| IL-12 | Cytokine, coefficient | 0.001  (-0.002 – 0.004) | 0.004  (-0.003 – 0.010) |
|  | Cytokine, p-value | 0.487 | 0.274 |
|  | qAlb, coefficient | -0.486  (-1.816 – 0.844) | 0.029  (-1.752 – 1.809) |
|  | qAlb, p-value | 0.469 | 0.975 |
|  | Interaction, coefficient | NA | -0.150  (-0.495 – 0.194) |
|  | Interaction, p-value | NA | 0.388 |
|  | R^2^ | 0.0129 | 0.0229 |
| IL-13 | Cytokine, coefficient | 0.017  (-0.004 – 0.038) | -0.008  (-0.047 – 0.031) |
|  | Cytokine, p-value | 0.114 | 0.684 |
|  | qAlb, coefficient | -9.755  (-25.813 – 6.303) | -101.029  (-222.954 – 20.897) |
|  | qAlb, p-value | 0.230 | 0.103 |
|  | Interaction, coefficient | NA | 2.372  (-0.769 – 5.512) |
|  | Interaction, p-value | NA | 0.137 |
|  | R^2^ | 0.0460 | 0.0743 |
| IFN-gamma | Cytokine, coefficient | -0.021  (-0.089 – 0.047) | 0.019  (-0.149 – 0.186) |
|  | Cytokine, p-value | 0.543 | 0.824 |
|  | qAlb, coefficient | -22.854  (-68.835 – 23.127) | 2.552  (-105.815 – 110.919) |
|  | qAlb, p-value | 0.325 | 0.963 |
|  | Interaction, coefficient | NA | -3.270  (-15.885 – 9.345) |
|  | Interaction, p-value | NA | 0.607 |
|  | R^2^ | 0.0177 | 0.0213 |
| TNF-alpha | Cytokine, coefficient | 0.009  (-0.011 – 0.030) | 0.005  (-0.031 – 0.041) |
|  | Cytokine, p-value | 0.356 | 0.795 |
|  | qAlb, coefficient | -0.959  (-4.511 – 2.593) | -4.475  (-27.093 – 18.142) |
|  | qAlb, p-value | 0.592 | 0.695 |
|  | Interaction, coefficient | NA | 0.404  (-2.160 – 2.968) |
|  | Interaction, p-value | NA | 0.755 |
|  | R^2^ | 0.0137 | 0.0150 |

**Supplementary Table 6** – Association of cytokine levels in cerebrospinal fluid (CSF) and plasma with outcome variables. Univariable logistic regression results shown for death. Ordinal logistic regression results shown for modified Rankin Scale (MRS) and SAH Outcome Tool (SAHOT) without Bonferroni correction. 0.10 > p > 0.05 in orange. p<0.05 in red.

| **Univariable regression** | | **Death (OR)** | **MRS (OR)** | | | | **SAHOT (OR)** | | |
| --- | --- | --- | --- | --- | --- | --- | --- | --- | --- |
|  |  |  | **Day 7** | **Day 28** | **Day 90** | **Day 180** | **Day 28** | **Day 90** | **Day 180** |
| CSF IL-1beta | OR/ coefficient (95% CI) | 1.491  (1.027 – 2.166) | 1.379 (1.113 – 1.707) | 1.261 (1.037 – 1.534) | 1.170 (0.962 – 1.422) | 1.191 (0.984 – 1.440) | 1.188 (0.989 – 1.428) | 1.048 (0.863 – 1.274) | 1.059 (0.871 – 1.287) |
|  | P-value | 0.036 | 0.003 | 0.020 | 0.116 | 0.073 | 0.066 | 0.634 | 0.564 |
| CSF IL-2 | OR/ coefficient (95% CI) | 1.223  (0.846 – 1.767) | 1.396 (1.107 – 1.761) | 1.162 (0.960 – 1.408) | 1.071 (0.883 – 1.300) | 1.010 (0.835 – 1.223) | 1.115 (0.925 – 1.344) | 1.041 (0.862 – 1.258) | 1.056 (0.878 -1.270) |
|  | P-value | 0.285 | 0.005 | 0.123 | 0.485 | 0.914 | 0.255 | 0.677 | 0.566 |
| CSF IL-4 | OR/ coefficient (95% CI) | 1.051  (0.988 – 1.119) | 1.065 (1.014 – 1.119) | 1.058 (1.015 – 1.103) | 1.026 (0.980 – 1.073) | 1.040 (0.996 – 1.086) | 1.029 (0.992 – 1.067) | 1.021 (0.979 – 1.064) | 1.020 (0.975 – 1.066) |
|  | P-value | 0.117 | 0.012 | 0.008 | 0.276 | 0.073 | 0.130 | 0.333 | 0.388 |
| CSF IL-6 | OR/ coefficient (95% CI) | 1.000 (1.000 – 1.000) | 1.000 (1.000 – 1.000) | 1.000 (1.000 – 1.000) | 1.000 (1.000 – 1.000) | 1.000 (1.000 – 1.000) | 1.000 (1.000 – 1.000) | 1.000 (1.000 – 1.000) | 1.000 (1.000 – 1.000) |
|  | P-value | 0.059 | 0.010 | 0.004 | 0.272 | 0.016 | 0.146 | 0.070 | 0.191 |
| CSF IL-8 | OR/ coefficient (95% CI) | 1.000 (1.000 – 1.001) | 1.000 (1.000 – 1.001) | 1.000 (1.000 – 1.001) | 1.000 (1.000 – 1.000) | 1.000 (1.000 – 1.000) | 1.000 (1.000 – 1.000) | 1.000 (1.000 – 1.000) | 1.000 (1.000 – 1.000) |
|  | P-value | 0.178 | 0.001 | 0.011 | 0.040 | 0.073 | 0.024 | 0.453 | 0.350 |
| CSF IL-10 | OR/ coefficient (95% CI) | 1.012  (0.937 – 1.092) | 0.993 (0.954 – 1.034) | 0.990 (0.950 – 1.032) | 0.944 (0.890 – 1.002) | 0.990 (0.951 – 1.031) | 0.988 (0.951 – 1.026) | 0.948 (0.889 – 1.011) | 0.963 (0.907 – 1.023) |
|  | P-value | 0.766 | 0.731 | 0.630 | 0.057 | 0.631 | 0.516 | 0.104 | 0.221 |
| CSF IL-12 | OR/ coefficient (95% CI) | 1.046  (0.990 – 1.011) | 1.051 (1.009 – 1.095) | 1.059 (1.022 – 1.097) | 1.027 (0.990 – 1.066) | 1.038 (1.001 – 1.076) | 1.033 (1.001 – 1.066) | 1.017 (0.982 – 1.053) | 1.015 (0.977 – 1.054) |
|  | P-value | 0.110 | 0.018 | 0.002 | 0.159 | 0.046 | 0.044 | 0.343 | 0.443 |
| CSF IL-13 | OR/ coefficient (95% CI) | 1.043  (1.004 – 1.084) | 1.032 (1.011 – 1.055) | 1.021 (1.001 – 1.042) | 1.016 (0.997 – 1.035) | 1.010 (0.992 – 1.029) | 1.009 (0.991 – 1.028) | 1.010 (0.991 – 1.031) | 1.009 (0.990 – 1.028) |
|  | P-value | 0.032 | 0.003 | 0.042 | 0.094 | 0.262 | 0.311 | 0.300 | 0.373 |
| CSF IFN-gamma | OR/ coefficient (95% CI) | 1.003  (0.959 – 1.050) | 1.000 (0.980 – 1.020) | 1.003 (0.984 – 1.022) | 1.004 (0.984 – 1.023) | 0.995 (0.976 – 1.015) | 1.005 (0.987 – 1.024) | 1.003 (0.983 – 1.024) | 0.999 (0.978 – 1.019) |
|  | P-value | 0.885 | 0.994 | 0.771 | 0.718 | 0.624 | 0.571 | 0.759 | 0.896 |
| CSF TNF-alpha | OR/ coefficient (95% CI) | 1.188  (1.040 – 1.358) | 1.074 (0.986 – 1.171) | 1.092 (0.990 – 1.205) | 1.095 (1.003 – 1.195) | 1.052 (0.962 – 1.150) | 1.086 (0.995 – 1.185) | 1.049 (0.962 – 1.144) | 1.047 (0.959 – 1.143) |
|  | P-value | 0.011 | 0.103 | 0.078 | 0.043 | 0.269 | 0.064 | 0.279 | 0.305 |
| Plasma IL-1beta | OR/ coefficient (95% CI) | 105.206  (0.372 – 29787.88) | 1.754  (0.088 – 35.008) | 0.443  (0.024 – 8.362) | 1.576  (0.078 – 31.977) | 5.703 (0.314 – 103.516) | 0.344 (0.020 – 5.999) | 5.933 (0.310 – 113.696) | 6.926 (0.382 – 125.477) |
|  | P-value | 0.106 | 0.713 | 0.587 | 0.767 | 0.239 | 0.464 | 0.237 | 0.190 |
| Plasma IL-2 | OR/ coefficient (95% CI) | 0.928  (0.219 – 3.936) | 1.609  (0.773 – 3.351) | 1.245 (0.831 – 1.866) | 1.216 (0.805 – 1.837) | 0.891 (0.579 – 1.372) | 1.122 (0.746 – 1.688) | 1.156 (0.765 – 1.749) | 1.113 (0.742 – 1.670) |
|  | P-value | 0.920 | 0.204 | 0.289 | 0.352 | 0.601 | 0.581 | 0.491 | 0.606 |
| Plasma IL-4 | OR/ coefficient (95% CI) | 760676.7  (0.000 – 2 x 10^15^) | 0.351  (0.000 – 3806.618) | 0.105 (0.000 – 1119.673) | 7.888 (0.001 – 96485.91) | 0.310 (0.000 – 2941.969) | 0.004 (0.000 – 54.550) | 305.497 (0.008 – 11700000) | 20.843 (0.000 – 870698) |
|  | P-value | 0.221 | 0.825 | 0.634 | 0.667 | 0.802 | 0.257 | 0.288 | 0.576 |
| Plasma IL-6 | OR/ coefficient (95% CI) | 1.415 (1.132 – 1.771) | 1.232  (1.097 – 1.383) | 1.168 (1.069 – 1.276) | 1.142 (1.042 – 1.251) | 1.206 (1.092 – 1.331) | 1.148 (1.058 – 1.244) | 1.095 (1.016 – 1.181) | 1.133 (1.027 – 1.249) |
|  | P-value | 0.002 | < 0.001 | 0.001 | 0.004 | < 0.001 | 0.001 | 0.018 | 0.012 |
| Plasma IL-8 | OR/ coefficient (95% CI) | 1.052  (1.006 – 1.100) | 1.042 (1.009 – 1.075) | 1.042 (1.011 – 1.073) | 1.023 (0.993 – 1.055) | 1.043 (1.012 – 1.075) | 1.025 (0.994 – 1.058) | 1.012 (0.980 – 1.046) | 1.017 (0.986 – 1.048) |
|  | P-value | 0.025 | 0.012 | 0.008 | 0.133 | 0.007 | 0.113 | 0.462 | 0.286 |
| Plasma IL-10 | OR/ coefficient (95% CI) | 1.102  (0.846 – 1.436) | 0.957 (0.796 – 1.150) | 1.029 (0.860 – 1.231) | 0.953 (0.796 – 1.141) | 0.954 (0.769 – 1.184) | 1.018 (0.866 – 1.198) | 1.037 (0.876 – 1.228) | 0.981 (0.829 – 1.161) |
|  | P-value | 0.472 | 0.636 | 0.757 | 0.597 | 0.668 | 0.828 | 0.673 | 0.826 |
| Plasma IL-12 | OR/ coefficient (95% CI) | 34.866  (1.021 – 1190.484) | 0.870 (0.086 – 8.753) | 2.195 (0.190 – 25.364) | 5.722 (0.624 – 52.464) | 11.306 (1.349 – 94.746) | 6.185 (0.711 – 53.787) | 19.809 (2.1.02 – 186.649) | 57.232 (5.005 – 654.382) |
|  | P-value | 0.049 | 0.906 | 0.529 | 0.123 | 0.025 | 0.099 | 0.009 | 0.001 |
| Plasma IL-13 | OR/ coefficient (95% CI) | 1.283  (0.967 – 1.703) | 0.981 (0.848 – 1.135) | 1.057 (0.908 – 1.231) | 1.034 (0.882 – 1.213) | 1.044 (0.895 – 1.218) | 1.052 (0.898 – 1.231) | 1.008 (0.845 – 1.203) | 1.076 (0.875 – 1.322) |
|  | P-value | 0.084 | 0.797 | 0.474 | 0.677 | 0.585 | 0.531 | 0.925 | 0.488 |
| Plasma IFN-gamma | OR/ coefficient (95% CI) | 0.987  (0.822 – 1.185) | 0.936  (0.878 – 0.999) | 0.988 (0.930 – 1.050) | 1.024  (0.957 – 1.096) | 0.989 (0.930 – 1.051) | 1.014 (0.954 – 1.078) | 1.043 (0.974 – 1.116) | 1.029 (0.962 – 1.099) |
|  | P-value | 0.886 | 0.046 | 0.698 | 0.488 | 0.719 | 0.650 | 0.228 | 0.406 |
| Plasma TNF-alpha | OR/ coefficient (95% CI) | 1.911  (0.366 – 9.977) | 0.483 (0.218 – 1.071) | 0.653 (0.300 – 1.419) | 0.744 (0.328 – 1.690) | 1.046 (0.481 – 2.276) | 0.713 (0.324 – 1.571) | 0.836 (0.362 – 1.931) | 0.687 (0.301 – 1.569) |
|  | P-value | 0.443 | 0.073 | 0.282 | 0.480 | 0.910 | 0.402 | 0.675 | 0.373 |

**Supplementary Figure 1** – Association of cytokines in the (A) cerebrospinal fluid (CSF) and (B) plasma of patients with subarachnoid haemorrhage with death, delayed cerebral ischaemia (DCI), modified Rankin Scale (mRS), and SAH Outcome Tool (SAHOT) variables after adjusting for the effects of cytokines within the same compartment with p<0.1 on univariable analysis. White > 0.1; 0.1 > Purple > 0.05; Black < 0.05.

**Appendix S1 – Sensitivity Analyses of Regression Models for Cytokines**

Multivariable regression was conducted for each outcome measure and each individual cytokine adjusting for blood volume. Among CSF cytokines, the significant associations were between CSF IL-8 and mRS score at day 7, CSF IL-12 and mRS score at day 28, CSF IL-13 and mRS score at day 7, and CSF TNF-α and death. Among plasma cytokines, only plasma IL-6 was significantly associated with all outcomes at all time points: death (OR 1.415 [95% CI: 1.104 – 1.812; p = 0.006]) mRS day 7 (OR 1.248 [95% CI: 1.102 – 1.412; p<0.001]), mRS day 28 (OR 1.147 [95% CI: 1.052 – 1.252; p = 0.002]), mRS day 90 (OR 1.127 [95% CI: 1.029 – 1.235; p = 0.010]), mRS day 180 (OR 1.195 [95% CI: 1.083 – 1.318; p<0.001]), SAHOT day 28 (OR 1.133 [95% CI: 1.044 – 1.230; p = 0.003]), SAHOT day 90 (OR 1.088 [95% CI: 1.007 – 1.176; p = 0.033]), and SAHOT day 180 (OR 1.118 [95% CI: 1.015 – 1.231; p = 0.023]). Among the other plasma cytokines, the only other significant associations were between plasma IL-12 and mRS score at day 180 and SAHOT scores at days 90 and 180.

Multivariable regression was conducted for each outcome measure and each individual cytokine adjusting for WFNS grade. Among CSF cytokines, the significant associations were between CSF IL-2 and mRS score at day 7, CSF IL-8 and mRS score at day 7, CSF IL-12 and mRS score at day 28, CSF IL-13 and death, and CSF TNF-α and death. Among plasma cytokines, only plasma IL-6 was significantly associated with all outcomes at all time points: death (OR 1.415 [95% CI: 1.107 – 1.809; p = 0.006]), mRS day 7 (OR 1.156 [95% CI: 1.035 – 1.292; p = 0.010]), mRS day 28 (OR 1.128 [95% CI: 1.048 – 1.213; p = 0.001]), mRS day 90 (OR 1.119 [95% CI: 1.034 – 1.211; p = 0.005]), mRS day 180 (OR 1.171 [95% CI: 1.063 – 1.289; p = 0.001]), SAHOT day 28 (OR 1.118 [95% CI: 1.039 – 1.203; p = 0.003]), SAHOT day 90 (OR 1.086 [95% CI: 1.010 – 1.168; p = 0.027]), and SAHOT day 180 (OR 1.129 [95% CI: 1.024 – 1.245; p = 0.015]). Among the other plasma cytokines, the only other significant associations were between plasma IL-8 and mRS score at day 180 and death, plasma IL-12 and death, mRS score at day 180 and SAHOT scores at days 90 and 180, and plasma IL-13 and death.

Multivariable models were created that assessed the association between each outcome and cytokines that were p<0.1 on univariable analysis, CT blood volume and WFNS grade. For example, no cytokine was significantly associated with death. The AIC of this model was 26.217. The model was simplified sequentially until the best AIC was achieved: 21.022. The predictors of death in this model were CSF IL-1, CSF IL-13, plasma IL-6, plasma IL-8, and CT blood volume; no cytokine was significantly associated with death. The model was simplified sequentially further until all predictors were significantly associated with the outcome. The AIC of this model was 23.913. The predictor of death in this model was only plasma IL-6 (p = 0.002). To take another example, only plasma IL-6 (OR 1.156 [95% CI: 1.036 – 1.290; p = 0.009]) was significantly associated with mRS 180 score. The AIC of this model was 279.964. The model was simplified sequentially until the best AIC was achieved: 269.993. The predictors of mRS 180 score in this model were CSF IL-6, plasma IL-6, and CT blood volume; only plasma IL-6 was significantly associated with mRS 180 score (OR 1.174 [95% CI: 1.060 – 1.300; p = 0.002]). The model was simplified sequentially further until all predictors were significantly associated with the outcome. The AIC of this model was 303.844. The predictor of mRS 180 score in this model was plasma IL-6 (p<0.001) and CT blood volume (p = 0.007).

**Supplementary Figure 2 –** a) A scree plot of the principal components for cerebrospinal fluid (CSF) cytokines. b) Heat map of the eigenvectors that form each component of the CSF cytokine principal components. Green = magnitude > 0.3; Yellow = magnitude > 0.1; Red = magnitude < 0.1.

**A**

**B**

| **Variable** | **Component 1** | **Component 2** | **Component 3** |
| --- | --- | --- | --- |
| **CSF IL-1β** | 0.3877 | 0.1344 | 0.3029 |
| **CSF IL-2** | 0.3449 | 0.1771 | -0.1755 |
| **CSF IL-4** | 0.3471 | -0.4032 | -0.0391 |
| **CSF IL-6** | 0.3311 | -0.3872 | 0.0021 |
| **CSF IL-8** | 0.2620 | 0.4476 | -0.0288 |
| **CSF IL-10** | 0.1343 | 0.0302 | 0.8961 |
| **CSF IL-12** | 0.3430 | -0.3762 | -0.0671 |
| **CSF IL-13** | 0.3444 | 0.2702 | -0.2134 |
| **CSF IFN-γ** | 0.2971 | -0.1969 | -0.1288 |
| **CSF TNF-α** | 0.2990 | 0.4285 | -0.0739 |

**Supplementary Figure 3 –** a) A scree plot of the principal components for plasma cytokines. b) Heat map of the eigenvectors that form each component of the plasma cytokine principal components. Green = magnitude > 0.3; Yellow = magnitude > 0.1; Red = magnitude < 0.1.

**A**

**B**

| **Variable** | **Component 1** | **Component 2** | **Component 3** | **Component 4** |
| --- | --- | --- | --- | --- |
| **Plasma IL-1β** | 0.2596 | 0.5220 | -0.0888 | -0.0995 |
| **Plasma IL-2** | 0.0081 | 0.4985 | 0.1021 | -0.2888 |
| **Plasma IL-4** | 0.2003 | 0.4219 | 0.1355 | 0.3758 |
| **Plasma IL-6** | 0.3984 | 0.1051 | -0.2790 | -0.1741 |
| **Plasma IL-8** | 0.4007 | -0.2829 | -0.3974 | 0.0146 |
| **Plasma IL-10** | 0.0490 | 0.2079 | -0.5357 | 0.6103 |
| **Plasma IL-12** | 0.4169 | 0.1234 | 0.1297 | -0.3777 |
| **Plasma IL-13** | 0.4681 | -0.2422 | 0.0841 | 0.0024 |
| **Plasma IFN-γ** | 0.1614 | 0.0827 | 0.5886 | 0.4291 |
| **Plasma TNF-α** | 0.3895 | -0.2932 | 0.2651 | 0.1890 |

**Supplementary Table 7** – Association of principal components of cytokine levels in CSF and plasma with outcome variables. Univariable logistic regression results are shown for death and delayed cerebral ischaemia (DCI). Ordinal logistic regression results are shown for modified Rankin Scale (MRS) and SAH Outcome Tool (SAHOT). Multivariable regression adjusts for the effects of all principal components with p < 0.1 on univariable analysis, World Federation of Neurosurgical Societies (WFNS) and blood volume 0.10 > p > 0.05 in orange. p < 0.05 in red.

| **Univariable regression** | | **Death (OR)** | **MRS (OR)** | | | | **SAHOT (OR)** | | |
| --- | --- | --- | --- | --- | --- | --- | --- | --- | --- |
|  |  |  | **Day 7** | **Day 28** | **Day 90** | **Day 180** | **Day 28** | **Day 90** | **Day 180** |
| CSF Principal Component 1 | OR/ coefficient (95% CI) | 1.584 (1.038 – 2.417) | 1.378 (1.129 – 1.680) | 1.303 (1.083 – 1.568) | 1.165 (0.969 – 1.400) | 1.165 (0.969 – 1.400) | 1.186 (1.000 – 1.406) | 1.099 (0.916 – 1.320) | 1.088 (0.905 – 1.308) |
|  | P-value | 0.033 | 0.002 | 0.005 | 0.104 | 0.104 | 0.051 | 0.311 | 0.367 |
| CSF Principal Component 2 | OR/ coefficient (95% CI) | 1.231 (0.648 – 2.338) | 1.111 (0.831 – 1.485) | 0.938 (0.711 – 1.239) | 1.146 (0.854 – 1.537) | 0.974 (0.721 – 1.316) | 1.038 (0.800 – 1.346) | 0.974 (0.733 – 1.295) | 1.020 (0.756 – 1.376) |
|  | P-value | 0.525 | 0.476 | 0.654 | 0.365 | 0.862 | 0.780 | 0.857 | 0.899 |
| CSF Principal Component 3 | OR/ coefficient (95% CI) | 0.885 (0.227 – 3.441) | 0.861 (0.619 – 1.196) | 0.859 (0.612 – 1.206) | 0.582 (0.334 – 1.013) | 0.937 (0.672 – 1.308) | 0.873 (0.641 – 1.189) | 0.595 (0.313 – 1.131) | 0.708 (0.416 – 1.205) |
|  | P-value | 0.860 | 0.371 | 0.380 | 0.055 | 0.703 | 0.389 | 0.113 | 0.203 |
| Plasma Principal Component 1 | OR/ coefficient (95% CI) | 2.087  (1.156 – 3.765) | 1.103 (0.864 – 1.409) | 1.269 (0.960 – 1.677) | 1.295 (0.987 – 1.699) | 1.476 (1.129 – 1.930) | 1.257 (0.940 – 1.679) | 1.350 (1.020 – 1.786) | 1.469 (1.091 – 1.978) |
|  | P-value | 0.015 | 0.432 | 0.094 | 0.062 | 0.004 | 0.123 | 0.036 | 0.011 |
| Plasma Principal Component 2 | OR/ coefficient (95% CI) | 1.312 (0.685 – 2.513) | 1.130 (0.821 – 1.554) | 0.992 (0.736 – 1.336) | 1.151 (0.842 – 1.574) | 0.997 (0.734 – 1.354) | 0.965 (0.702 – 1.328) | 1.341 (0.966 – 1.861) | 1.255 (0.913 – 1.724) |
|  | P-value | 0.412 | 0.454 | 0.958 | 0.379 | 0.983 | 0.828 | 0.079 | 0.161 |
| Plasma Principal Component 3 | OR/ coefficient (95% CI) | 0.458 (0.219 – 0.962) | 0.532 (0.361 – 0.783) | 0.679 (0.466 – 0.989) | 0.931 (0.635 – 1.365) | 0.740 (0.516 – 1.062) | 0.852 (0.606 – 1.198) | 0.989 (0.702 – 1.394) | 0.962 (0.677 – 1.366) |
|  | P-value | 0.039 | 0.001 | 0.044 | 0.714 | 0.102 | 0.357 | 0.950 | 0.827 |
| Plasma Principal Component 4 | OR/ coefficient (95% CI) | 0.615 (0.237 – 1.595) | 0.626 (0.435 – 0.899) | 0.805 (0.575 – 1.127) | 0.792 (0.557 – 1.125) | 0.708 (0.489 – 1.024) | 0.822 (0.598 – 1.130) | 0.922 (0.651 – 1.306) | 0.757 (0.531 – 1.079) |
|  | P-value | 0.317 | 0.011 | 0.206 | 0.192 | 0.066 | 0.228 | 0.647 | 0.124 |
| **Multivariable regression** | | **Death (OR)** | **MRS (OR)** | | | | **SAHOT (OR)** | | |
|  |  |  | **Day 7** | **Day 28** | **Day 90** | **Day 180** | **Day 28** | **Day 90** | **Day 180** |
| CSF Principal Component 1 | OR/ coefficient (95% CI) | 1.429 (0.711 – 2.871) | 1.089 (0.866 – 1.369) | 1.077 (0.881 – 1.316) | NA | NA | 1.030 (0.857 – 1.238) | NA | NA |
|  | P-value | 0.316 | 0.466 | 0.471 | NA | NA | 0.751 | NA | NA |
| CSF Principal Component 2 | OR/ coefficient (95% CI) | NA | NA | NA | NA | NA | NA | NA | NA |
|  | P-value | NA | NA | NA | NA | NA | NA | NA | NA |
| CSF Principal Component 3 | OR/ coefficient (95% CI) | NA | NA | NA | 0.606 (0.353 – 1.043) | NA | NA | NA | NA |
|  | P-value | NA | NA | NA | 0.070 | NA | NA | NA | NA |
| Plasma Principal Component 1 | OR/ coefficient (95% CI) | 3.048 (0.974 – 9.541) | NA | 1.313 (0.971 – 1.774) | 1.335 (0.997 – 1.788) | 1.525 (1.156 – 2.012) | NA | 1.311 (0.998 – 1.722) | 1.442 (1.050 – 1.981) |
|  | P-value | 0.056 | NA | 0.076 | 0.053 | 0.003 | NA | 0.051 | 0.024 |
| Plasma Principal Component 2 | OR/ coefficient (95% CI) | NA | NA | NA | NA | NA | NA | 1.415 (1.013 – 1.975) | NA |
|  | P-value | NA | NA | NA | NA | NA | NA | 0.042 | NA |
| Plasma Principal Component 3 | OR/ coefficient (95% CI) | 0.599 (0.189 – 1.891) | 0.743 (0.485 – 1.138) | 0.928 (0.635 – 1.355) | NA | NA | NA | NA | NA |
|  | P-value | 0.382 | 0.172 | 0.698 | NA | NA | NA | NA | NA |
| Plasma Principal Component 4 | OR/ coefficient (95% CI) | NA | 0.573 (0.348 – 0.943) | NA | NA | 0.734 (0.513 – 1.048) | NA | NA | NA |
|  | P-value | NA | 0.028 | NA | NA | 0.089 | NA | NA | NA |

**Supplementary Figure 4** – Association of principal components (PCs) of cytokines in the plasma in patients with subarachnoid haemorrhage with death, delayed cerebral ischaemia (DCI), modified Rankin Scale (mRS), and SAH Outcome Tool (SAHOT), after adjusting for the effects of all other PCs within the same compartment. White > 0.1; 0.1 > Purple > 0.05; Black < 0.05.

**Appendix S2 – Sensitivity Analyses of Regression Models for Principal Components**

Multivariable regression was conducted for each outcome measure and each principal component adjusting for blood volume. Among CSF principal components, there were no significant associations with any outcome. Among plasma principal components, the significant associations were between the first plasma principal component and death, mRS score at day 180 and SAHOT scores at days 90 and 180, the second plasma principal component and SAHOT score at day 90, the third plasma principal component and DCI, and the fourth plasma principal component and mRS score at day 7.

Multivariable regression was conducted for each outcome measure and each principal component adjusting only for WFNS grade. Among CSF principal components, there were no significant associations with any outcome. Among plasma principal components, the significant associations were between the first plasma principal component and death, DCI, mRS score at day 180 and SAHOT scores at days 90 and 180, the third plasma principal component and death, DCI and mRS score at day 7, and the fourth plasma principal component and mRS score at day 7.

After adjusting for WFNS grade and CT blood volume, the association between the first plasma principal component and mRS 180 was weaker than the association between plasma IL-6 and mRS 180, and the model fit worse (AIC 311.562 vs 304.874).

Multivariable models were created that assessed the association between each outcome and principal components that were p<0.1 on univariable analysis, CT blood volume and WFNS grade. For example, no principal component was significantly associated with death. The AIC of this model was 31.073. The model was simplified sequentially until the best AIC was achieved: 28.514. The predictors of death in this model were the first CSF principal component and the first plasma principal component; only the first plasma principal component was significantly associated with death (p = 0.024). The model was simplified sequentially further until all predictors were significantly associated with the outcome. The AIC of this model was 33.305. The predictor of death in this model was only the first plasma principal component (p = 0.015). To take another example, only the first plasma principal component (OR 1.525 [95% CI: 1.156 – 2.012 p = 0.003]) was significantly associated with mRS 180 score. The AIC of this model was 310.649. This was the model with the best AIC. The model was simplified sequentially further until all predictors were significantly associated with the outcome. The AIC of this model was 312.776. The predictor of mRS 180 score in this model was the first plasma principal component (p = 0.005) and CT blood volume (p = 0.003).

**Supplementary Figure 5** **– An example of a pathway analysis model**

**mRS 180**

**Plasma IL-6**

**Blood Volume**

**Supplementary Table 8 – Characteristics of studies evaluating the association of cytokines with outcomes in patients with subarachnoid haemorrhage**

| **Study ID** | **Number of patients** | **Study design** | **Cytokines (/their receptors) measured** | **Compartments sampled** | **Timing of sampling** | **Trend in levels** | **Outcomes examined (and timepoints)** | **Associations reported** |
| --- | --- | --- | --- | --- | --- | --- | --- | --- |
| **Mathiesen et al., 1993** (1) | 12 SAH | Prospective cohort study | IL-6 | CSF, serum | Days 1, 3, 6, 9 | IL-6 showed a marked increase in CSF (up to 300-fold) after SAH. This was not paralleled by a systemic increase of IL-6 in plasma | Delayed ischemic deficit (DID) at unspecified time point | Mean CSF IL-6 in patients with DID increased significantly |
| **Kikuchi et al., 1995** (2) | 7 SAH | Prospective cohort study | IL-1α, IL-1β, IL-6, IL-8, TNF-α | CSF, serum | Days 3-4, 7-8, 13-14 | The concentrations of IL-6 and IL-8 - but not the other cytokines - in the CSF after SAH are much higher than in the serum | DID at unspecified time point | Not reported |
| **Hirashima et al., 1997** (3) | 21 SAH | Prospective cohort study | IL-1β, IL-6, TNF-α | Plasma | Days 0, 4, 5, 9, 10, 14 | IL-6 concentrations increased within the first 4 days after SAH and remained elevated up to 14 days, IL-1β transiently increased between 5-9 days after SAH, and TNF-α remained unchanged | Delayed ischemic neurological deficits (DIND) within 14 days | IL-6 levels significantly increased in patients suffering from DIND |
| **Mathieson et al., 1997** (4) | 22 SAH 10 controls | Prospective cohort study | IL-1Ra, TNF-α | CSF | Days 1, 2, 3, 4, 5, 6, 7, 8, 9 , 10, 11, 12 | Significant increases in IL-1Ra and TNFα were detected during days 4 through 10 in patients suffering from SAH who eventually had a poor outcome. Patients with good outcomes and control patients had low levels of these cytokines. | DID within 12 days, Glasgow Outcome Scale (GOS) at unspecified time point | Patients with an unfavourable outcome had marked increases in IL-1Ra levels between Days 3 and 10. Patients with GOS scores of 3 or less or who experienced an episode of DID had IL-1Ra levels that were higher than 1000 pg/ml at some time point, whereas no other patient had levels greater than 500 pg/ml |
| **McKeating et al., 1997** (5) | 10 SAH | Prospective cohort study | IL-1β, IL-6, IL-8, TNF-α | Serum | Hours 12, 24, 48, 96 | Not reported | Not reported | Increased jugular venous serum concentrations of IL-6 relative to arterial concentrations after SAH, |
| **Gaetani et al., 1998** (6) | 31 SAH 10 controls | Prospective cohort study | IL-6, IL-8 | CSF | Days 0-3, 10+ | CSF levels of IL-6 and IL-8 were higher in samples obtained in the earlier time period | Symptomatic vasospasm as an inpatient | Significant increase of IL-6 in patients who developed vasospasm |
| **Osuka et al., 1998** (7) | 24 SAH 9 controls | Prospective cohort study | IL-1β, IL-6, IL-8 | CSF, serum | Days 0, 1, 2, 3, 5, 7, 11, 14 | CSF concentrations of IL-6 and IL-8 in patients with SAH were thousands of times higher than in serum or in control CSF samples throughout, but IL-6 and IL-8 serum concentrations were only higher than controls in the acute period | Symptomatic vasospasm between days 5 and 8 | Levels of IL-6 in CSF of the patients with symptomatic vasospasm revealed a significant excess on day 5 and 7. Concentrations of IL-8 in CSF of patients with symptomatic vasospasm also were significantly higher on day 5 |
| **Gruber et al., 2000** (8) | 44 SAH | Prospective cohort study | sTNFR-I, IL-1ra, IL-6 | CSF, serum | Days 0–2, 3–5, 6–8, 9–11, 12–14 | In the CSF of SAH patients, sTNFR-I, IL-1ra, and IL-6 levels were significantly higher than those of the non-SAH control group. This was also true for the mean serum concentrations of sTNFR-I and IL-1ra, whereas IL-6 serum levels were not significantly elevated. | Multiple Organ Dysfunction Score on admission; GOS at 6 months | The post-SAH IL-1ra and IL-6 levels correlated with worse GOS scores |
| **Fassbender et al., 2001** (9) | 35 SAH 20 controls | Prospective cohort study | IL-1β, IL-6, TNF-α | CSF, plasma | Days 1, 2, 3, 5, 7, 9, 11 | In SAH, concentrations of IL-1β and IL-6 were significantly lower in plasma than in CSF | Vasospasm between days 1 -11; GOS at unspecified time point | CSF IL-6 concentration at day 5 were significantly increased in patients with poor (GOS 1-2) compared with those with moderate (GOS3-5) outcome. Vasospasm was associated with increased CSF concentrations of IL-1β, IL-6, and TNF-α |
| **Kwon et al., 2001** (10) | 19 SAH 12 controls | Prospective cohort study | IL-1β, IL-6, TNF-α | CSF | Day 0 | Significantly increased CSF IL-6 level after SAH compared with controls | DID, GOS at unspecified time point | IL-6 level 4-fold higher in patient with DID compared with patient without. No correlation with outcome on GOS |
| **Schoch et al., 2007** (11) | 64 SAH | Prospective cohort study | IL-6 | CSF | Days 1, 2, 3, 4, 5, 6, 7, 8, 9 , 10, 11, 12, 13, and 14 | Mean CSF IL-6 values were 500-fold greater in SAH patients compared with expected normal levels | Vasospasm, delayed cerebral ischemia (DCI), Rankin scale by/at discharge | Significant correlation between IL-6 CSF and DCI, but no significant correlation with Rankin scale |
| **Nakahara et al., 2009** (12) | 39 SAH 13 controls | Prospective cohort study | IL-6, IL-8, TNF-α | CSF | Days 3, 7, 14 | CSF IL-6 levels graphically peaked at day 7 | GOS at 3 months | In the unfavourable outcome group, IL-6, IL-8, and TNF-α were significantly increased. |
| **Sarrafzadeh et al., 2010** (13) | 38 SAH | Prospective cohort study | IL-6 | CSF, plasma, ECF | Days 0, 1, 2, 3, 4, 5, 6, 7, 8, 9 , 10 | Highest IL-6 levels were measured in CSF followed by ECF, then plasma | DCI or neurological deficit during admission, GOS at 6 and 12 months | Cerebral, but not plasma IL-6, levels were predictive for the development of ischaemic deficits |
| **Graetz et al., 2010** (14) | 24 SAH | Prospective cohort study | IL-6 | CSF, plasma, ECF | Days 0, 1, 2, 3, 4, 5, 6, 7, 8, 9 , 10 | Highest IL-6 levels were measured in CSF followed by ECF, then plasma. Patients with higher intracranial pressure had higher IL-6 levels. | DIND within 10 days, GOS at 3 and 6 months | In the unfavourable outcome group, IL-6 levels were significantly increased. |
| **Muroi et al., 2011** (15) | 99 SAH 20 controls | Prospective cohort study | IL-6 | Plasma | Days 0, 1, 2, 3, 4, 5, 6, 7, 8, 9 , 10 | Aneurysmatic SAH patients had significantly higher IL-6 values compared with peri- mesencephalic SAH patients | Vasospasm between days 4 and 14, GOS at 3 months | Higher IL-6 levels in patients who had vasospasm |
| **Ni et al., 2011** (16) | 46 SAH | Prospective cohort study | IL-6 | CSF | Days 1, 2, 3, 5, 7 | The median level of IL-6 in CSF is greater than 400pg/ml at all time points | Vasospasm within 14 days | Patients with vasospasm had higher median values of IL-6 CSF on all days |
| **Chou et al., 2012** (17) | 52 SAH | Prospective cohort study | IL-6, TNF-α | Serum | Days 0–1, 2–3, 4–5, 6–8, 10–14 | Not reported | Vasopasm between days 6-8, modified Rankin (mRS) score at 3 and 6 months | Global elevation of TNFα levels over time (post-SAH days 0–14) is significantly associated with poor mRS outcome at 3 months after adjusting for SAH clinical severity and age |
| **Chou et al., 2013** (18) | 29 SAH | Prospective cohort study | IL-2, IL-4, IL-5, IL17A | CSF | Not reported | Not reported | Vasopasm on day 7, mRS score at 3 and 6 months | CSF IL-4 showed strongest trend towards association with good outcome at 3-month and at 6-months |
| **McMahon et al., 2013** (19) | 149 SAH | Case control study | IL-1Ra, IL-6 | Plasma | Days 0, 1, 2, 3, 4, 5, 6, 7, 8, 9 , 10, 11, 12, 13 | A rise in plasma cytokine levels generally occurred following angiography | DCI within 15 days, GOS at 6 months | The rate of change of IL-6 was associated with DCI |
| **Muroi et al., 2013** (20) | 138 SAH | Prospective cohort study | IL-6 | Plasma | Days 0, 1, 2, 3, 4, 5, 6, 7, 8, 9 , 10, 11, 12, 13, 14 | IL-6 levels tended to be high at the beginning (day 3) with a discrete decrease in the following 2 days, followed by a second increase beginning at day 7, with a peak around day 10 to 11. | DIND at unspecified time point, GOS at 3 months | Higher IL-6 levels in the early phase (days 3-7) were associated with the occurrence of DIND, and unfavourable outcomes |
| **Helbok et al., 2015** (21) | 26 SAH | Prospective cohort study | IL-6 | ECF | Hours 0, 12, 24, 36, 48, 72, 96, 120, 144 | IL-6 levels were highest in the first 36 hours | Vasospasm, DCI during admission, mRS at 3 months | ECF IL-6 levels were higher in patients with poor 3-month mRS |
| **Höllig et al., 2015** (22) | 81 SAH | Prospective cohort study | IL-6 | CSF, serum | Day 0-1 | Not reported | DIND at unspecified time point, mRS at discharge and 6 months | Higher early IL-6 serum levels after aSAH was associated with poor outcome at discharge |
| **Höllig et al., 2015** (23) | 53 SAH | Prospective cohort study | IL-6 | Serum | Days 0, 1, 4, 7, 10, 14 | The time course of serum IL-6 differed by the grade of the severity of the SAH | mRS at discharge and 6 months | Higher IL-6 levels across time in the group with an unfavourable outcome. |
| **Kao et al., 2015** (24) | 53 SAH | Prospective cohort study | IL-6 | Plasma | Day of emmolisation | Not reported | mRS at 30 days | Patients with high l IL-6 levels were more likely to have poor neurological outcomes |
| **Schallner et al., 2015** (25) | 11 SAH Unknown number of controls | Not reported | IL-1β, IL-6, IL-8, Il-10, TNF-α | CSF | Days 1, 7 | All CSF cytokines had levels higher than controls. IL-1β, IL-8, and TNF-α levels at day 7 were greater than their levels at day 1 | Not reported | Not reported |
| **Tang et al., 2015** (26) | 58 SAH 20 controls | Prospective cohort study | IL-6 | Plasma | Days 1, 3-5, 8-10 | Plasma IL-6 levels were highest on day 1 | Vasospasm, DCI during admission, GOS at unspecified time point | The concentration of plasma IL-6 was significantly higher in patients who had vasospasm, DCI, or a poor outcome |
| **Zhou et al., 2015** (27) | 43 SAH 4 controls | Prospective cohort study | IL-1β, TNF-α | CSF | Days 1-3, 4-7, 8+ | CSF IL-1β and TNF-α were increased in SAH patients | GOS and mRS at discharge | Not reported |
| **Wu et al., 2016** (28) | 57 SAH 65 controls | Case control study | IL-6, TNF-α | CSF | Day 2 | Higher IL-6 and TNF-α levels in CSF of SAH patients compared to healthy controls | Vasospasm during admission | Patients who developed vasospasm had elevated CSF IL-6 |
| **Niwa et al., 2016** (29) | 10 SAH 5 controls | Prospective cohort study | IL-6, IP-10, MCP-1 | CSF | Days 1, 2, 3, 4, 5, 6, 7, 8, 9 , 10, 11, 12, 13, 14 | The concentration of IL-6 increased significantly during the acute stage of the disease, reaching a level more than 1000 times that of the control group, and then decreased gradually thereafter | GOS at 3 months | The peak concentration of IL-6 increased significantly in poor outcome |
| **Chamling et al., 2017** (30) | 89 SAH | Prospective cohort study | IL-6 | Serum | Days 0, 1, 4, 7, 10, 14 | Not reported | DCI | Baseline levels of IL-6 was significantly associated with occurrence of DCI |
| **Chaudhry et al., 2017** (31) | 80 SAH 10 controls | Prospective cohort study | IL-6 | Serum | Days 1, 3, 5, 7, 9, 11, 13 | Serum IL-6 levels were found to be significantly elevated in SAH patients presenting with higher Hunt and Hess grades, increasing age, and both intraventricular and intracerebral hemorrhage. IL-6 was also significantly raised in patients who developed seizures and chronic hydrocephalus | Vasospasm, DIND during admission | Higher IL-6 levels in patient who developed vasospasm and DIND |
| **Chen et al., 2017** (32) | 102 SAH 102 controls | Prospective cohort study | IL-6, TNF-α | Serum | Day 0-1 | Not reported | Symptomatic vasospasm, extended GOS (GOSE) at 6 months | Serum concentrations of IL-6 and TNF-α was increased in patients suffering from an unfavorable outcome at 6 months |
| **Kiiski et al., 2017** (33) | 47 SAH | Prospective cohort study | IL-6 | Plasma | Hours 0, 12, 24, 48, 72, 96, 120 | Not reported | DCI during admission, mRS at 6 months | Plasma IL-6 was not associated with outcomes |
| **Lenski et al., 2017** (34) | 63 SAH | Prospective cohort study | IL-6 | CSF, serum | Not reported | Not reported | Vasospasm, GOS at discharge from ICU | Patients who developed vasospasm had elevated CSF IL-6 |
| **Savarraj et al., 2017** (35) | 45 SAH | Retrospective observational study | CCL2, CCL5, CCL7, CCL11, CCL22, CX3CL1, CXCL1P1, MIP-1α, MIP-1β, IL-1A, IL-1R1, IL-4, IL-5, IL-6, IL-17a, IL-2, IL-3, IL-7, IL-8, IL-9, IL-10, IL-13, IL-15, PDGF-AA, PDGF-AB/BB, EGF, FGF-2, CSF3, CSF2, TNF-α, TNF-β, VEGFA, IL-12p40, IL-12p70, sCD40L, IL-1b, interferon-inducible protein 10, IFNG, IFN-α2, FLT3L, TGF-α | Plasma | Day 0, 1-2, 3-5, 6-8 | IL-6, CCL2, CCL11, CSF3, IL-8, IL-10, CX3CL1, TNF-α were also significantly elevated | DCI during admission | High PDGF-AB/BB in days 1-2 was observed in participants who subsequently developed DCI |
| **Schiefecker et al., 2017** (36) | 26 SAH | Prospective cohort study | IL-6 | ECF | Unable to extract | Unable to extract | Unable to extract | Unable to extract |
| **Zhou et al., 2017** (37) | 27 SAH | Prospective cohort study | IL-2, IL-4, IL-10, IFN-γ | Plasma | Day 0, and days 1, 3, and 6 after surgical operation | Following surgery, the levels of IL-4, IFN-γ, and IL-2 were significantly increased, whereas the level of IL-10 in plasma was decreased. The levels of IL-4, IFN-γ, and IL-2 showed a steady decrease after day 3 after surgery, and the values were close to that of the admission levels on day 6 post-surgery | GOS at 3 months, 6 months, and 12 months | Not reported |
| **Zhong et al., 2017** (38) | 89 SAH 12 controls | Prospective cohort study | IL-1β, IL-2, IL-6, IL-8, IL-10 | Serum | Day 0 | Not reported | DCI during admission, GOS at 6 months | Higher levels of IL-6, IL-10 assoicated with DCI and a poor outcome in patients with aneurysmal SAH. |
| **Ďuriš. et al., 2018** (39) | 47 SAH | Prospective cohort study | IL-1β, IL-6, TNF-α | CSF, plasma | Days 1, 2, 3, 4, 5 | CSF IL-6 levels increased after SAH greater than plasma levels of IL-6. There was a change in CSF IL-6 rate of increase at 3 days | Vasospasm during admission, GOS at 3 months | Significant association between IL-6 and GOS for both overall levels of IL-6 and their dynamics |
| **Gong et al., 2018** (40) | 175 SAH 175 controls | Prospective cohort study | IL-33 | Serum | Day 0 | Serum IL-33 concentrations in patients were significantly higher than those in controls, and markedly higher in non-survivors than in survivors within 6 months | GOS at 6 months | Serum IL-33 concentrations >741.3 ng/ml yielded a sensitivity of 75.8% and a specificity of 79.6% in discriminating patients at risk of 6-month unfavorable outcome |
| **Lv et al., 2018** (41) | 81 SAH Unknown number of controls | Prospective cohort study | IL-1β, IL-18, TNF-α | CSF | Days 1-3, 4-6, 7-9 | The levels of IL-1β, IL-18, and TNF-α in the CSF were significantly increased in aSAH patients | mRS at 6 months | The levels of CSF IL-1β, IL-18, and TNF-α in each period of time were significantly correlated with poor outcome |
| **Righy et al., 2018** (42) | 10 SAH | Prospective cohort study | IL-1β, IL-2, IL-4, IL-5, IL-6, IL-7, IL-8, IL- 10, IL-12, IL-13, IL-17, IFN-γ, G-CSF, MCP-1, MIP-1, TNF-α | CSF, plasma | Days 1, 2, 3 | Not reported | Not reported | Not reported |
| **Savarraj et al., 2018** (43) | 60 SAH 100 controls | Retrospective observational study | CCL2, CCL5, CCL7, CCL11, CCL22, CX3CL1, CXCL1P1, MIP-1α, MIP-1β, IL-1A, IL-1R1, IL-4, IL-5, IL-6, IL-17a, IL-2, IL-3, IL-7, IL-8, IL-9, IL-10, IL-13, IL-15, PDGF-AA, PDGF-AB/BB, EGF, FGF-2, CSF3, CSF2, TNF-α, TNF-β, VEGFA, IL-12p40, IL-12p70, sCD40L, IL-1b, interferon-inducible protein 10, IFNG, IFN-α2, FLT3L, TGF-α | Plasma | Day 1 after surgery | Not reported | DCI during admission, mRS at discharge | Plasma IL-6, IL-8, IL-10, IP-10, CCL2, and TNF-α levels were significantly higher in patients with poorer outcomes |
| **Savarraj et al., 2018** (44) | 71 SAH | Prospective observational study | IL-1, IL-1α, IL-1Ra, IL-4, IL-5, IL-6, IL-10, IL-17a, IFN-γ, MCP1, MIP1α, MIP1β, TNF-α | Serum | Day 0-2 | IL-6 was elevated in patients with global cerebral ischemia, in patients with higher subarachnoid hemorrhage early brain edema score and higher Hunt-Hess-score | DCI during admission, mRS at discharge | Plasma IL-6 is independently associated with mRS at discharge |
| **Wang et al., 2018** (45) | 43 SAH 23 controls | Prospective observational study | IL-6 | Serum | Days 1, 4, 7, 10 | Expression levels of IL-6 were elevated with the increase of Hunt-Hess grades | Vasospasm during admission | Patients who developed vasospasm had elevated serum IL-6 |
| **Ahn et al., 2019** (46) | 60 SAH | Prospective cohort study | CCL2, CCL5, CCL7, CCL11, CCL22, CX3CL1, CXCL1P1, MIP-1α, MIP-1β, IL-1A, IL-1R1, IL-4, IL-5, IL-6, IL-17a, IL-2, IL-3, IL-7, IL-8, IL-9, IL-10, IL-13, IL-15, PDGF-AA, PDGF-AB/BB, EGF, FGF-2, CSF3, CSF2, TNF-α, TNF-β, VEGFA, IL-12p40, IL-12p70, sCD40L, IL-1b, interferon-inducible protein 10, IFNG, IFN-α2, FLT3L, TGF-α | Serum | Day 0, 1-2, 3-5, 6-8 | Not reported | DCI during admission, mRS at discharge and at 3 months | Median levels of PDGF-ABBB (particularly at days 1-2) and CCL5 (particularly at days 1-2, 3-5) were consistently higher, and median levels of IP-10 (particularly at days 0, 1-2) and MIP-1α (particularly at days 6-8) were consistently lower in the DCI group compared to the no-DCI group. Median levels of IL-6 (particularly at days 1-2) and MCP-1 (particularly at days 6-8) were consistently higher in subjects with poor functional outcome than those with good functional outcome |
| **Al-Tamimi et al., 2019** (47) | 43 SAH 11 controls | Prospective cohort study | IL-1α, IL-1β, IL-4, IL-6, IL-8, IL-10, IL-15, IL-17, IL-18, TNF-α, MCP-1, VEGF | CSF, plasma | Days 1-3, 5, 7, 9 | CSF concentrations were significantly higher in aSAH patients compared to controls for all mediators (except for IL-1α) at all time points, except day 9. Plasma IL-1α levels in the aSAH group were significantly lower than in controls on days 3, 5 and 7. Plasma IL-6 and IL-8 levels were significantly higher on days 5 and 7. Cytokine concentrations were consistently higher in CSF apart from IL to 1α and IL-18 (which were higher in plasma) | DIND at unspecified time point, mRS at 10 days and 6 months | When comparing DIND and non-DIND patients, only IL-4 was statistically significantly higher in the DIND group. Day 3 plasma IL-6 levels predicted poor mRS outcome at six months, although this association was lost in the second analysis incorporating Fisher grade, WFNS grade and age. |
| **Matsumoto et al., 2019** (48) | 14 SAH 7 controls | Retrospective observational study | IL-6 | CSF, serum | Hours 3 , 48 , 96 , 144 , 192, 240 | The CSF IL-6 concentration was lowest within the first 48 hours | Vasospasm during admission | The CSF IL-6 concentration increased after DSA in patients who had vasospasm evident on DSA |
| **Rasmussen et al., 2019** (49) | 90 SAH | Prospective cohort study | IL-6, IL-8, IL-10, IFNγ, TNF-α | Plasma | Days 2-4, 7-9 | Not reported | Vasospasm on day 7-9, DCI during admission, GOS at 3 months | Plasma levels of IL-6, IL-8, IL-10, IFNγ, and TNF-α were not associated with DCI, vasospasm, or GOS at 3 months |
| **Ridwan et al., 2019** (50) | 50 SAH | Prospective cohort study | IL-6 | CSF, serum | Days 3, 7, 14, 21 | CSF IL-6 levels were significantly higher than serum IL-6 levels | mRS, GOS, Short Form health survey questionnaire, the Beck Depression Inventory, and the Daily Fatigue Impact Scale at 6 and 12 months. | Higher IL-6 serum levels were associated with reduced fatigue |
| **Vlachogiannis et al., 2019** (51) | 44 SAH | Prospective cohort study | IL-6 | CSF, plasma | Day 0-1, 4, 10 | In CSF, the initial IL-6 levels on day 1 were followed by significantly increased IL-6 values on day 4 that had decreased on day 10, but still remained significantly higher than the day 1 values. In plasma, IL-6 values were above the reference interval already from day 1, remained stable until day 4, and decreased on day 10. | Vasospasm during admission, GOS at 12 months | No significant association between IL-6 levels and outcomes |
| **Chaudhry et al., 2020** (52) | 76 SAH | Prospective cohort study | IL-10 | Serum | Days 1, 7 | Serum IL-10 levels were significantly higher in SAH patients on post-SAH day 1 and day 7 compared to the control patients | Vasospasm, DCI during admission, GOS, mRS at discharge | There was a significant elevation in serum IL-10 levels on day 7 in SAH patients who developed cerebral vasospasm. Serum IL-10 levels were significantly higher on both day 1 and day 7 post-SAH in patients with poor clinical outcome |
| **Coulibaly et al., 2020** (53) | 10 SAH | Prospective cohort study | IL-1α, IL-1β, IL-1ra, IL-2, IL-4, IL-5, IL-6, IL-8, IL-10, IL-12 p70, IL-13, IL-16, IL-17, IL-23, IL-27, CCL2, CCL3, CCL4, CCL5, CXCL10, CXCL11, CXCL12 | CSF | Day 3 | Not reported | Vasospasm during admission, mRS at 90 days | Five cytokines were elevated in patients with poor outcome: IL-1α, TNFα, TREM-1, IL-2, and IL-17 |
| **Moraes et al., 2020** (54) | 39 SAH 56 controls | Prospective cohort study | TH1, TH2 and TH17 cytokines | CSF, serum | Days 1-3, 4-6 | Serum and CSF levels of IL-6, TNF-α, IL-17A, IL-10 and IL-2 in early and delayed phase of aSAH patients were increased compared to controls | Vasospasm between days 1-14 | IL-17A serum level in the early phase of aSAH patients was significantly increased in patients who developed vasospasm |
| **Gusdon et al., 2020** (55) | 57 SAH | Prospective cohort study | CCL2, CCL5, CCL7, CCL11, CCL22, CX3CL1, CXCL1P1, MIP-1α, MIP-1β, IL-1A, IL-1R1, IL-4, IL-5, IL-6, IL-17a, IL-2, IL-3, IL-7, IL-8, IL-9, IL-10, IL-13, IL-15, PDGF-AA, PDGF-AB/BB, EGF, FGF-2, CSF3, CSF2, TNF-α, TNF-β, VEGFA, IL-12p40, IL-12p70, sCD40L, IL-1b, interferon-inducible protein 10, IFNG, IFN-α2, FLT3L, TGF-α | Serum | Days 0-1, 1-2, 3-5, 6-8 | Compared with HP 1-1 and 1-2, subjects encoding HP 2-2 had elevated levels of the following cytokines at all time points: FLT3L, IFNγ, IL-17A, TGFα, and VEGF-A. Elevations were also seen at some time points for IL-8, CSF2, FGF2, IL-7, IL-12p70, and TNFα. | Vasospasm during admission, mRS at discharge and 3 months | Not reported |
| **Yang et al., 2020** (56) | 201 SAH | Prospective cohort study | IL-6 | Serum | Day 1 | Not reported | Vasospasm during admission, DCI within 3 months | Patients with higher levels of IL-6 were more likely to suffer from DCI |
| **Bjerkne et al., 2021** (57) | 58 SAH | Prospective cohort study | IL-1Ra, IL-6, TNF-α | Serum | Days 0-1, 10 | Not reported | DCI during admission, GOSE at 1 year | IL‐1Ra levels were significantly associated with poor outcome |
| **Ridwan et al., 2021** (58) | 82 SAH | Prospective cohort study | IL-6 | CSF, serum | Days 0-3, 4-14 | Not reported | Vasospasm, DCI within 21 days, mRS at discharge | Significant correlation between CSF IL-6 and DCI. Baseline CSF IL-6 levels following SAH predictors of DCI. No correlations between individual serum IL-6 peak levels and DCI. High CSF IL-6 levels corresponded to worse outcome on modified Rankin scale |
| **Luo et al., 2022** (59) | 165 SAH 86 controls | Prospective cohort study | IL-1, IL-2, IL-4, IL-5, IL-6, IL-8, IL-10, IL-12p70, IL-17, IFN-α, IFN-γ, and TNF-α | Serum | Not reported | Serum IL-1β, IL-5, IL-6, IL-8, IL-10, IFN-γ, and TNF-α levels were significantly higher in the mild group than in the severe group. | mRS at 90 days | After propensity score matching, IL-2, IL-6, IL-8, IL-10, and TNF-α levels were higher in patients with worse mRS scores. Serum IL-6 levels had the largest AUC for predicting poor mRS outcomes at 90 days |
| **Xu et el., 2022** (60) | 54 SAH | Prospective cohort study | IL-1β, IL-6, IL-8, TNF-α | CSF | Days 1, 3, 5, and 7 | IL-1β levels in CSF peaked at>60 pg/ml on day 1, then decreased on days 3, 5, and 7.CSF levels of IL-6 were > 2000 pg/ml on day 1, and remained high on days 3 and 5, subsequently decreasing on day 7. IL-8 levels peaked at>3000 pg/ml at day 3 after aSAH, subsiding on days 5 and 7. TNF-α levels increased between days 1 and 5, peaking at>75 pg/ml on day 5 and then declining abruptly | Not reported | Not reported |
| **Hoadley et al., 2023** (61) | 11 SAH 22 controls | Prospective cohort study | IL-1β, IL-4, IL-6, IL-8, IL-17, IFN-γ, TNF-α | Plasma | Days 0, 3 | Not reported | Not reported | Not reported |
| **Fischer et al., 2023** (62) | 66 SAH | Prospective cohort study | IL-6, IL-17, IL-23, IL-10, CCL5) | Plasma | Day 0 - 1 | Not reported | Vasospasm during admission, GOS, mRS at discharge | IL-6, IL-10, and vasospasm were correlated with one another |
| **Vlachogiannis et al., 2023** (63) | 29 SAH | Prospective cohort study | CCL2 (or MCP-1), CCL3, CCL4, CCL7 (or MCP-3), CCL8 (or MCP-2), CCL11 (or Eotaxin), CCL13 (or MCP-4), CCL19, CCL20, CCL23, CCL25, CCL28, CXCL1, CXCL5, CXCL6, CXCL8 (or IL-8), CXCL9, CXCL10, CXCL11 and CX3CL1 | CSF | Days 1, 4, 10 | Four chemokines (CCL3, CCL4, CCL11 and CCL20) showed early peak and decreasing values. CCL28 showed a statistically significant middle peak. The majority of the chemokines (i.e., 11/20; notably, almost all members of the C-X-C chemokine family except CXCL5) showed increasing trends throughout the observation period and late peaks on day 10 | DCI during admission, GOS at 12 months | Significantly higher day 10 mean values were observed in patients with poor outcome for chemokines CCL2, CCL4, CCL7, CCL11, CCL13, CCL19, CCL20, CXCL1, CXCL5, CXCL6 and CXCL8. Patients with DCI had significantly higher day 4 values of CXCL5 |

**Appendix S3 – Narrative summary of the association of cytokines with outcomes in patients with subarachnoid haemorrhage from existing studies.**

Thirteen studies have measured IL-6 in both CSF and plasma/serum, and have reported significantly higher levels of IL-6 in the CSF (1,2,50,51,54,7–9,13,14,39,47,48). Although several existing studies have presented graphical trends of small series of CSF or plasma/serum cytokine levels with time that would be consistent with our findings, few have presented them together and none have discussed the implications of any differences between the compartments. Two small series of SAH patients (n = 38 and n = 12) displayed figures consistent with IL-6 levels being highest in the plasma in the first few days after SAH, and between days 6 – 8 in the CSF (1,13). Another small series of SAH patients (n = 35) presented figures consistent with TNF-α peaking between days 5 to 9 in the CSF (9). Two previous studies produced findings which differed in some aspects from ours, and this may be potentially explained by differences in study design. The first study was a small series (n = 24) which found that levels of cytokines were highest in the plasma in the first few days after SAH – similar to our study – but CSF cytokines peaked at ictus and around day 7 (7), though steroid treatment may have altered the temporal pattern of CSF cytokines. The second study found that IL-6 and IL-8 levels in the CSF were significantly greater within 3 days after SAH, compared to day 10 (6), but the samples from day 3 and 10 were obtained from two different populations. With a larger sample size, paired CSF/serum sampling and a panel of ten cytokines in the same cohort, we provide definitive evidence that the temporal behaviour of cytokine levels is distinctly different in plasma and CSF.

No studies to date have directly assessed the correlation between plasma and CSF cytokines and how this correlation is related to BBB integrity.

Our study also found that CSF cytokine levels were related to WFNS grade and blood load. This is congruent with what is described in the existing literature. Studies have shown that CSF IL-6 and TNF-α levels increased with the severity of the SAH as defined by the Hunt & Hess classification (8,28). We now show that not only these, but five other CSF cytokines correlate with WFNS. We show that CSF cytokines are directly related to the volume of the SAH, but plasma cytokines do not relate to either WFNS or SAH volume.

The relationship between cytokines and clinical outcome is difficult to piece together from the existing literature, for various reasons. The most robust evidence exists for IL-6, but often studying plasma and CSF in isolation. Several studies have shown plasma IL-6 is associated with unfavourable functional outcome (mRS and Glasgow Outcome Scale) after aSAH on both univariable and multivariable analyses (20,22,57,58,23,24,26,31,38,39,46,47). Of these studies, four have additionally reported on the association between CSF IL-6 and functional outcomes (22,39,47,58), and of those only two report on both plasma and CSF IL-6 associations with late outcomes at 6 months or later (22,47). Of these two studies, one found no association between CSF IL-6 levels and outcomes at 6 months (22). The other study found that day 3 plasma IL-6 levels were associated with worse mRS outcomes at 6 months, but this association was lost after adjusting for Fisher grade, WFNS grade, and age (47). Additionally, several studies have reported only on the association between CSF IL-6 and outcomes (4,10–12,18,27,29,41,53,63). These studies reported conflicting results. The discrepancies may arise from the timing of CSF sampling or timing of outcome measurement. As seen in our study, CSF cytokines may be associated with outcome in the short-term, but not in the long-term. Furthermore, the studies that found an association between CSF IL-6 levels and unfavourable outcomes in the long-term only did so on univariable analysis (8,12,39,47,58). Given CSF cytokines are strongly associated with blood volume and WFNS grade, it is important to correct for these baseline variables.

Even less data is available for other cytokines. CSF TNF-α levels were reported to be higher in patients with poorer outcomes in a series of 22 patients (4), though the timing of outcome assessment is not specified, and adjustment was not made for blood volume, WFNS grade or multiple comparisons. To highlight the importance of adjusting for multiple comparisons, an abstract reporting on a series of 29 patients found that out of 42 markers only IL-4 was associated with 6 month outcome (18), but this did not survive correction for multiple comparisons. Similar considerations apply to studies of other serum/plasma cytokines (e.g., IL-8) that have found an association between that cytokine and poor outcome (59,63).

Far more data exists regarding the relationship of cytokines and vasospasm/DCI than for functional outcome, mostly supporting an association, though some are contradictory. One study showed that the average levels of CSF IL-6 and TNF- α on day 2 after admission were greater in patients with vasospasm (28). Another study found higher levels of CSF IL-6 within 72 hours from ictus was associated with vasospasm (6). In contrast, we did not find a significant association between CSF IL-6 levels at day 7 and TCDs. Taken together with our study, these findings could be interpreted as demonstrating that CSF IL-6 levels at an earlier stage predict vasospasm, but no longer do so at day 7. This is supported by another study, which found CSF IL-6 on day 6 and 7 was not associated with vasospasm, but CSF from days 2-4 was (day 4 having the strongest association with vasospasm) (11). It is possible a similar scenario applies for DCI given Sarrafzadeh et al. reported that CSF IL-6, but not plasma IL-6 levels were indicative of the development of DCI (13), although it is not possible to be sure as they did not report which day post-ictus they used for their analysis and they collected CSF samples from multiple timepoints.

Since timing of CSF sampling seems an attractive explanation for divergent observations, we performed subgroup analyses using the serial samples obtained from EVDs. This analysis considered a similarly selected severely unwell patient group with EVDs. Despite this there remained no relationships between CSF cytokines and vasospasm and/or DCI.

Our finding of an association with plasma IL-6 and DCI is in keeping with seven other studies which found no association between this cytokine and DCI (13,21,33,35,46,49,58). However, some discrepancies still exist. For example, one study found day 0 serum IL-6 was inversely associated with DCI, but that this was not seen when the analysis was performed with maximum IL-6 levels (30). This may suggest that plasma IL-6 in the short-term may not be deleterious, but a sustained rise in IL-6 may lead to pathological effects. Consistent with this hypothesis, a study found that it took until day 3 for serum IL-6 levels to be significantly associated with DCI (31), and another study found that it was the rate of decrease of plasma IL-6 that is relevant, with patients going on to have DCI having a smaller decrease (19).

**References**

1. Mathiesen T, Andersson B, Loftenius A, Von Holst H. Increased interleukin-6 levels in cerebrospinal fluid following subarachnoid hemorrhage. J Neurosurg [Internet]. 1993 [cited 2023 Nov 5];78(4):562–7. Available from: https://pubmed.ncbi.nlm.nih.gov/8450329/

2. Kikuchi T, Okuda Y, Kaito N, Abe T. Cytokine production in cerebrospinal fluid after subarachnoid haemorrhage. Neurol Res [Internet]. 1995 [cited 2023 Nov 5];17(2):106–8. Available from: https://pubmed.ncbi.nlm.nih.gov/7609845/

3. Hirashima Y, Nakamura S, Endo S, Kuwayama N, Naruse Y, Takaku A. Elevation of platelet activating factor, inflammatory cytokines, and coagulation factors in the internal jugular vein of patients with subarachnoid hemorrhage. Neurochem Res [Internet]. 1997 [cited 2023 Nov 5];22(10):1249–55. Available from: https://pubmed.ncbi.nlm.nih.gov/9342729/

4. Mathieson T, Edner G, Ulfarsson E, Andersson B. Cerebrospinal fluid interleukin-1 receptor antagonist and tumor necrosis factor-alpha following subarachnoid hemorrhage. J Neurosurg [Internet]. 1997 Aug 1 [cited 2023 Mar 16];87(2):215–20. Available from: https://thejns.org/view/journals/j-neurosurg/87/2/article-p215.xml

5. Mckeating EG, Andrews PJD, Signorini DF, Mascia L. Transcranial cytokine gradients in patients requiring intensive care after acute brain injury. Br J Anaesth [Internet]. 1997 [cited 2023 Nov 5];78(5):520–3. Available from: https://pubmed.ncbi.nlm.nih.gov/9175965/

6. Gaetani P, Tartara F, Pignatti P, Tancioni F, Rodriguez Y Baena R, De Benedetti F. Cisternal CSF levels of cytokines after subarachnoid hemorrhage. Neurol Res [Internet]. 1998 [cited 2023 Nov 7];20(4):337–42. Available from: https://pubmed.ncbi.nlm.nih.gov/9618698/

7. Osuka K, Suzuki Y, Tanazawa T, Hattori K, Yamamoto N, Takayasu M, et al. Interleukin-6 and development of vasospasm after subarachnoid haemorrhage. Acta Neurochir (Wien) [Internet]. 1998 [cited 2023 Nov 5];140(9):943–51. Available from: https://link.springer.com/article/10.1007/s007010050197

8. Gruber A, Rössler K, Graninger W, Donner A, Illievich UM, Czech T. Ventricular cerebrospinal fluid and serum concentrations of sTNFR-I, IL-1ra, and IL-6 after aneurysmal subarachnoid hemorrhage. J Neurosurg Anesthesiol [Internet]. 2000 [cited 2023 Mar 23];12(4):297–306. Available from: https://pubmed.ncbi.nlm.nih.gov/11147377/

9. Fassbender K, Hodapp B, Rossol S, Bertsch T, Schmeck J, Schütt S, et al. Inflammatory cytokines in subarachnoid haemorrhage: association with abnormal blood flow velocities in basal cerebral arteries. J Neurol Neurosurg Psychiatry [Internet]. 2001 Apr 1 [cited 2022 Nov 4];70(4):534–7. Available from: https://pubmed.ncbi.nlm.nih.gov/11254783/

10. Kwon KY, Jeon BC. Cytokine levels in cerebrospinal fluid and delayed ischemic deficits in patients with aneurysmal subarachnoid hemorrhage. J Korean Med Sci [Internet]. 2001 [cited 2024 Feb 22];16(6):774. Available from: /pmc/articles/PMC3054809/?report=abstract

11. Schoch B, Regel JP, Wichert M, Gasser T, Volbracht L, Stolke D. Analysis of intrathecal interleukin-6 as a potential predictive factor for vasospasm in subarachnoid hemorrhage. Neurosurgery [Internet]. 2007 May [cited 2023 Apr 18];60(5):828–35. Available from: https://pubmed.ncbi.nlm.nih.gov/17460517/

12. Nakahara T, Tsuruta R, Kaneko T, Yamashita S, Fujita M, Kasaoka S, et al. High-mobility group box 1 protein in CSF of patients with subarachnoid hemorrhage. Neurocrit Care [Internet]. 2009 Jan 24 [cited 2023 Apr 18];11(3):362–8. Available from: https://link.springer.com/article/10.1007/s12028-009-9276-y

13. Sarrafzadeh A, Schlenk F, Gericke C, Vajkoczy P. Relevance of cerebral interleukin-6 after aneurysmal subarachnoid hemorrhage. Neurocrit Care [Internet]. 2010 Dec 20 [cited 2023 Mar 16];13(3):339–46. Available from: https://link.springer.com/article/10.1007/s12028-010-9432-4

14. Graetz D, Nagel A, Schlenk F, Sakowitz O, Vajkoczy P, Sarrafzadeh A. High ICP as trigger of proinflammatory IL-6 cytokine activation in aneurysmal subarachnoid hemorrhage. Neurol Res [Internet]. 2010 Sep 1 [cited 2023 Nov 6];32(7):728–35. Available from: https://pubmed.ncbi.nlm.nih.gov/19682408/

15. Muroi C, Bellut D, Coluccia D, Mink S, Fujioka M, Keller E. Systemic interleukin-6 concentrations in patients with perimesencephalic non-aneurysmal subarachnoid hemorrhage. J Clin Neurosci [Internet]. 2011 Dec [cited 2024 Feb 22];18(12):1626–9. Available from: https://pubmed.ncbi.nlm.nih.gov/22019436/

16. Ni W, Gu YX, Song DL, Leng B, Li PL, Mao Y. The relationship between IL-6 in CSF and occurrence of vasospasm after subarachnoid hemorrhage. Acta Neurochir Suppl [Internet]. 2011 [cited 2024 Feb 22];110(Pt 1):203–8. Available from: https://pubmed.ncbi.nlm.nih.gov/21116940/

17. Chou SHY, Feske SK, Atherton J, Konigsberg RG, De Jager PL, Du R, et al. Early elevation of serum tumor necrosis factor-α is associated with poor outcome in subarachnoid hemorrhage. J Investig Med [Internet]. 2012 [cited 2024 Feb 22];60(7):1054–8. Available from: https://pubmed.ncbi.nlm.nih.gov/22918199/

18. Chou SH, Elyaman W, Bradshaw EM, Secor EA, Suh S, Orent W, et al. Abstract TP426: Candidate CSF Cytokine and Chemokine Biomarkers for Vasospasm and Poor Outcome in Human Subarachnoid Hemorrhage. Stroke [Internet]. 2013 Feb [cited 2023 Nov 6];44(suppl_1). Available from: https://www.ahajournals.org/doi/abs/10.1161/str.44.suppl_1.atp426

19. McMahon CJ, Hopkins S, Vail A, King AT, Smith D, Illingworth KJ, et al. Original research: Inflammation as a predictor for delayed cerebral ischemia after aneurysmal subarachnoid haemorrhage. J Neurointerv Surg [Internet]. 2013 Nov [cited 2023 Nov 7];5(6):512. Available from: /pmc/articles/PMC3812893/

20. Muroi C, Hugelshofer M, Seule M, Tastan I, Fujioka M, Mishima K, et al. Correlation among systemic inflammatory parameter, occurrence of delayed neurological deficits, and outcome after aneurysmal subarachnoid hemorrhage. Neurosurgery [Internet]. 2013 Mar [cited 2023 Mar 23];72(3):367–75. Available from: https://pubmed.ncbi.nlm.nih.gov/23208059/

21. Helbok R, Schiefecker AJ, Beer R, Dietmann A, Antunes AP, Sohm F, et al. Early brain injury after aneurysmal subarachnoid hemorrhage: A multimodal neuromonitoring study. Crit Care [Internet]. 2015 Mar 9 [cited 2023 Jan 15];19(1):1–9. Available from: https://pubmed.ncbi.nlm.nih.gov/25887441/

22. Höllig A, Remmel D, Stoffel-Wagner B, Schubert GA, Coburn M, Clusmann H. Association of early inflammatory parameters after subarachnoid hemorrhage with functional outcome: A prospective cohort study. Clin Neurol Neurosurg [Internet]. 2015 Nov [cited 2023 Mar 23];138:177–83. Available from: https://pubmed.ncbi.nlm.nih.gov/26355810/

23. Höllig A, Thiel M, Stoffel-Wagner B, Coburn M, Clusmann H. Neuroprotective properties of dehydroepiandrosterone-sulfate and its relationship to interleukin 6 after aneurysmal subarachnoid hemorrhage: a prospective cohort study. Crit Care [Internet]. 2015 Dec 14 [cited 2023 Nov 21];19(1). Available from: /pmc/articles/PMC4462180/

24. Kao HW, Lee KW, Kuo CL, Huang CS, Tseng WM, Liu CS, et al. Interleukin-6 as a Prognostic Biomarker in Ruptured Intracranial Aneurysms. PLoS One [Internet]. 2015 Jul 15 [cited 2023 Jan 15];10(7). Available from: https://pubmed.ncbi.nlm.nih.gov/26176774/

25. Schallner N, Pandit R, LeBlanc R, Thomas AJ, Ogilvy CS, Zuckerbraun BS, et al. Microglia regulate blood clearance in subarachnoid hemorrhage by heme oxygenase-1. J Clin Invest [Internet]. 2015 Jul 1 [cited 2023 Jan 15];125(7):2609–25. Available from: https://pubmed.ncbi.nlm.nih.gov/26011640/

26. Tang Q-F, Lu S-Q, Zhao Y-M, Qian J-X. The changes of von willebrand factor/a disintegrin-like and metalloprotease with thrombospondin type I repeats-13 balance in aneurysmal subarachnoid hemorrhage. Int J Clin Exp Med [Internet]. 2015 [cited 2023 Nov 21];8(1):1342. Available from: /pmc/articles/PMC4358590/

27. Zhou C, Xie G, Wang C, Zhang Z, Chen Q, Zhang L, et al. Decreased progranulin levels in patients and rats with subarachnoid hemorrhage: a potential role in inhibiting inflammation by suppressing neutrophil recruitment. J Neuroinflammation [Internet]. 2015 Nov 2 [cited 2023 Jan 15];12(1). Available from: https://pubmed.ncbi.nlm.nih.gov/26527034/

28. Wu W, Guan Y, Zhao G, Fu XJ, Guo TZ, Liu YT, et al. Elevated IL-6 and TNF-α Levels in Cerebrospinal Fluid of Subarachnoid Hemorrhage Patients. 2016 Jul 1 [cited 2023 Jan 15];53(5):3277–85. Available from: https://pubmed.ncbi.nlm.nih.gov/26063595/

29. Niwa A, Osuka K, Nakura T, Matsuo N, Watabe T, Takayasu M. Interleukin-6, MCP-1, IP-10, and MIG are sequentially expressed in cerebrospinal fluid after subarachnoid hemorrhage. J Neuroinflammation [Internet]. 2016 Aug 30 [cited 2023 Jan 15];13(1). Available from: https://pubmed.ncbi.nlm.nih.gov/27576738/

30. Chamling B, Gross S, Stoffel-Wagner B, Schubert GA, Clusmann H, Coburn M, et al. Early Diagnosis of Delayed Cerebral Ischemia: Possible Relevance for Inflammatory Biomarkers in Routine Clinical Practice? World Neurosurg. 2017 Aug 1;104:152–7.

31. Chaudhry SR, Stoffel-Wagner B, Kinfe TM, Güresir E, Vatter H, Dietrich D, et al. Elevated Systemic IL-6 Levels in Patients with Aneurysmal Subarachnoid Hemorrhage Is an Unspecific Marker for Post-SAH Complications. Int J Mol Sci [Internet]. 2017 Dec 1 [cited 2023 Jan 15];18(12):2580. Available from: /pmc/articles/PMC5751183/

32. Chen YH, Cheng ZY, Shao LH, Shentu HS, Fu B. Macrophage migration inhibitory factor as a serum prognostic marker in patients with aneurysmal subarachnoid hemorrhage. Clin Chim Acta [Internet]. 2017 Oct 1 [cited 2023 Jan 15];473:60–4. Available from: https://pubmed.ncbi.nlm.nih.gov/28823650/

33. Kiiski H, Långsjö J, Tenhunen J, Ala-Peijari M, Huhtala H, Hämäläinen M, et al. Time-courses of plasma IL-6 and HMGB-1 reflect initial severity of clinical presentation but do not predict poor neurologic outcome following subarachnoid hemorrhage. eNeurologicalSci [Internet]. 2016 Mar 1 [cited 2024 Feb 22];6:55–62. Available from: https://pubmed.ncbi.nlm.nih.gov/29260012/

34. Lenski M, Huge V, Briegel J, Tonn JC, Schichor C, Thon N. Interleukin 6 in the Cerebrospinal Fluid as a Biomarker for Onset of Vasospasm and Ventriculitis After Severe Subarachnoid Hemorrhage. World Neurosurg [Internet]. 2017 Mar 1 [cited 2023 Jan 15];99:132–9. Available from: https://pubmed.ncbi.nlm.nih.gov/27931942/

35. Savarraj JPJ, Parsha K, Hergenroeder GW, Zhu L, Bajgur SS, Ahn S, et al. Systematic model of peripheral inflammation after subarachnoid hemorrhage. Neurology [Internet]. 2017 Apr [cited 2023 Jan 15];88(16):1535–45. Available from: /pmc/articles/PMC5395070/

36. Schiefecker AJ, Dietmann A, Beer R, Pfausler B, Lackner P, Kofler M, et al. Neuroinflammation is Associated with Brain Extracellular TAU-Protein Release After Spontaneous Subarachnoid Hemorrhage. Curr Drug Targets [Internet]. 2017 Feb 2 [cited 2023 Jan 15];18(12). Available from: https://pubmed.ncbi.nlm.nih.gov/26844567/

37. Jiang Y, Zhou Y, Peng Y, Zhang M. The Quantitative and Functional Changes of Postoperative Peripheral Blood Immune Cell Subsets Relate to Prognosis of Patients with Subarachnoid Hemorrhage: A Preliminary Study. World Neurosurg [Internet]. 2017 Dec 1 [cited 2023 Jan 15];108:206–15. Available from: https://pubmed.ncbi.nlm.nih.gov/28866066/

38. Zhong W, Zhang Z, Zhao P, Shen J, Li X, Wang D, et al. The Impact of Initial Systemic Inflammatory Response After Aneurysmal Subarachnoid Hemorrhage. Turk Neurosurg [Internet]. 2017 [cited 2023 Jan 15];27(3):346–52. Available from: https://pubmed.ncbi.nlm.nih.gov/27593784/

39. Ďuriš K, Neuman E, Vybíhal V, Juráå V, Gottwaldová J, Kýr M, et al. Early Dynamics of Interleukin-6 in Cerebrospinal Fluid after Aneurysmal Subarachnoid Hemorrhage. J Neurol Surg A Cent Eur Neurosurg [Internet]. 2018 Mar 1 [cited 2023 Nov 21];79(2):145–51. Available from: https://pubmed.ncbi.nlm.nih.gov/28869993/

40. Gong J, Zhu Y, Yu J, Jin J, Chen M, Liu W, et al. Increased serum interleukin-33 concentrations predict worse prognosis of aneurysmal subarachnoid hemorrhage. Clin Chim Acta [Internet]. 2018 Nov 1 [cited 2023 Jan 15];486:214–8. Available from: https://pubmed.ncbi.nlm.nih.gov/30102896/

41. Lv S yin, Wu Q, Liu J peng, Shao J, Wen L li, Xue J, et al. Levels of Interleukin-1β, Interleukin-18, and Tumor Necrosis Factor-α in Cerebrospinal Fluid of Aneurysmal Subarachnoid Hemorrhage Patients May Be Predictors of Early Brain Injury and Clinical Prognosis. World Neurosurg [Internet]. 2018 Mar 1 [cited 2023 Jan 15];111:e362–73. Available from: https://pubmed.ncbi.nlm.nih.gov/29277532/

42. Righy C, Turon R, De Freitas G, Japiassú AM, De Castro Faria Neto HC, Bozza M, et al. Hemoglobin metabolism by-products are associated with an inflammatory response in patients with hemorrhagic stroke. Rev Bras Ter intensiva [Internet]. 2018 Jan 1 [cited 2023 Jan 15];30(1):21–7. Available from: https://pubmed.ncbi.nlm.nih.gov/29742229/

43. Savarraj JP, McGuire MF, Parsha K, Hergenroeder G, Bajgur S, Ahn S, et al. Disruption of thrombo-inflammatory response and activation of a distinct cytokine cluster after subarachnoid hemorrhage. Cytokine [Internet]. 2018 Nov 1 [cited 2023 Jan 15];111:334–41. Available from: https://pubmed.ncbi.nlm.nih.gov/30269030/

44. Savarraj J, Parsha K, Hergenroeder G, Ahn S, Chang TR, Kim DH, et al. Early Brain Injury Associated with Systemic Inflammation After Subarachnoid Hemorrhage. Neurocrit Care [Internet]. 2018 Apr 1 [cited 2023 Jan 15];28(2):203–11. Available from: https://pubmed.ncbi.nlm.nih.gov/29043545/

45. Wang L, Gao Z. Expression of MMP-9 and IL-6 in patients with subarachnoid hemorrhage and the clinical significance. Exp Ther Med [Internet]. 2018 Feb 1 [cited 2024 Feb 22];15(2):1510. Available from: /pmc/articles/PMC5774465/

46. Ahn SH, Savarraj JPJ, Parsha K, Hergenroeder GW, Chang TR, Kim DH, et al. Inflammation in delayed ischemia and functional outcomes after subarachnoid hemorrhage. J Neuroinflammation [Internet]. 2019 Nov 11 [cited 2023 Jan 15];16(1). Available from: https://pubmed.ncbi.nlm.nih.gov/31711504/

47. Al-Tamimi YZ, Bhargava D, Orsi NM, Teraifi A, Cummings M, Ekbote U V., et al. Compartmentalisation of the inflammatory response following aneurysmal subarachnoid haemorrhage. Cytokine [Internet]. 2019 Nov 1 [cited 2023 Jan 15];123. Available from: https://pubmed.ncbi.nlm.nih.gov/31323526/

48. Matsumoto A, Nakamura T, Shinomiya A, Kawakita K, Kawanishi M, Miyake K, et al. Histidine-rich Glycoprotein Could Be an Early Predictor of Vasospasm after Aneurysmal Subarachnoid Hemorrhage. Acta Med Okayama [Internet]. 2019 [cited 2023 Jan 15];73(1):29–39. Available from: https://pubmed.ncbi.nlm.nih.gov/30820052/

49. Rasmussen R, Bache S, Stavngaard T, Møller K. Plasma Levels of IL-6, IL-8, IL-10, ICAM-1, VCAM-1, IFNγ, and TNFα are not Associated with Delayed Cerebral Ischemia, Cerebral Vasospasm, or Clinical Outcome in Patients with Subarachnoid Hemorrhage. World Neurosurg [Internet]. 2019 Aug 1 [cited 2024 Feb 22];128:e1131–6. Available from: https://pubmed.ncbi.nlm.nih.gov/31121365/

50. Ridwan S, Greschus S, Boström J, Barrera J, Esche J, Zur B, et al. Spontaneous Aneurysmal Subarachnoid Hemorrhage and Related Cortisol and Immunologic Alterations: Impact on Patients’ Health-related Quality of Life. J Neurol Surg A Cent Eur Neurosurg [Internet]. 2019 [cited 2024 Feb 22];80(5):371–80. Available from: https://pubmed.ncbi.nlm.nih.gov/31272120/

51. Vlachogiannis P, Hillered L, Khalil F, Enblad P, Ronne-Engström E. Interleukin-6 Levels in Cerebrospinal Fluid and Plasma in Patients with Severe Spontaneous Subarachnoid Hemorrhage. World Neurosurg [Internet]. 2019 Feb 1 [cited 2023 Jan 15];122:e612–8. Available from: https://pubmed.ncbi.nlm.nih.gov/30814021/

52. Chaudhry SR, Kahlert UD, Kinfe TM, Lamprecht A, Niemelä M, Hänggi D, et al. Elevated Systemic IL-10 Levels Indicate Immunodepression Leading to Nosocomial Infections after Aneurysmal Subarachnoid Hemorrhage (SAH) in Patients. Int J Mol Sci [Internet]. 2020 Mar 1 [cited 2024 Feb 22];21(5). Available from: https://pubmed.ncbi.nlm.nih.gov/32106601/

53. Coulibaly AP, Gartman WT, Swank V, Gomes JA, Ruozhuo L, DeBacker J, et al. RAR-Related Orphan Receptor Gamma T (RoRγt)-Related Cytokines Play a Role in Neutrophil Infiltration of the Central Nervous System After Subarachnoid Hemorrhage. Neurocrit Care [Internet]. 2020 Aug 1 [cited 2023 Jan 15];33(1):140–51. Available from: https://pubmed.ncbi.nlm.nih.gov/31768758/

54. Moraes L, Trias N, Brugnini A, Grille P, Lens D, Biestro A, et al. TH17/Treg imbalance and IL-17A increase after severe aneurysmal subarachnoid hemorrhage. J Neuroimmunol [Internet]. 2020 Sep 15 [cited 2023 Jan 15];346. Available from: https://pubmed.ncbi.nlm.nih.gov/32623101/

55. Gusdon AM, Savarraj J, Zhu L, Pandit PKT, Doré S, McBride DW, et al. Haptoglobin Genotype Affects Inflammation after Aneurysmal Subarachnoid Hemorrhage. Curr Neurovasc Res [Internet]. 2020 Dec 15 [cited 2023 Jan 15];17(5):652–9. Available from: https://pubmed.ncbi.nlm.nih.gov/33319684/

56. Yang X, Peng J, Pang J, Wan W, Zhong C, Peng T, et al. The Association Between Serum Macrophage Migration Inhibitory Factor and Delayed Cerebral Ischemia After Aneurysmal Subarachnoid Hemorrhage. Neurotox Res [Internet]. 2020 Feb 1 [cited 2023 Jan 15];37(2):397–405. Available from: https://pubmed.ncbi.nlm.nih.gov/31267487/

57. Bjerkne Wenneberg S, Odenstedt Hergès H, Svedin P, Mallard C, Karlsson T, Adiels M, et al. Association between inflammatory response and outcome after subarachnoid haemorrhage. Acta Neurol Scand [Internet]. 2021 Feb 1 [cited 2023 Jan 15];143(2):195–205. Available from: https://pubmed.ncbi.nlm.nih.gov/32990943/

58. Ridwan S, Grote A, Simon M. Interleukin 6 in cerebrospinal fluid is a biomarker for delayed cerebral ischemia (DCI) related infarctions after aneurysmal subarachnoid hemorrhage. Sci Rep [Internet]. 2021 Dec 1 [cited 2023 Jan 15];11(1):12. Available from: /pmc/articles/PMC7794326/

59. Luo C, Yao J, Bi H, Li Z, Li J, Xue G, et al. Clinical Value of Inflammatory Cytokines in Patients with Aneurysmal Subarachnoid Hemorrhage. Clin Interv Aging [Internet]. 2022 [cited 2023 May 2];17:615. Available from: /pmc/articles/PMC9056097/

60. Xu L, Wang W, Lai N, Tong J, Wang G, Tang D. Association between pro-inflammatory cytokines in cerebrospinal fluid and headache in patients with aneurysmal subarachnoid hemorrhage. J Neuroimmunol [Internet]. 2022 May 15 [cited 2024 Feb 22];366. Available from: https://pubmed.ncbi.nlm.nih.gov/35279426/

61. Hoadley ME, Galea J, Singh N, Hulme S, Ajao DO, Rothwell N, et al. The role of cortisol in immunosuppression in subarachnoid haemorrhage. Eur J Med Res [Internet]. 2023 Dec 1 [cited 2024 Feb 22];28(1). Available from: https://pubmed.ncbi.nlm.nih.gov/37644600/

62. Fischer I, Chaudhry SR, Hänggi D, Muhammad S. Clustering of serum biomarkers involved in post-aneurysmal subarachnoid hemorrhage (aSAH) complications. Neurosurg Rev [Internet]. 2023 Dec 1 [cited 2024 Feb 22];46(1):63. Available from: /pmc/articles/PMC9981718/

63. Vlachogiannis P, Hillered L, Enblad P, Ronne-Engström E. Elevated levels of several chemokines in the cerebrospinal fluid of patients with subarachnoid hemorrhage are associated with worse clinical outcome. PLoS One [Internet]. 2023 Mar 1 [cited 2023 Nov 22];18(3). Available from: https://pubmed.ncbi.nlm.nih.gov/36893189/
